# Methodological description of clinical research data collection through electronic medical records in a center participating in an international multicenter study

**DOI:** 10.31744/einstein_journal/2019AE4791

**Published:** 2019-09-16

**Authors:** Veronica Neves Fialho Queiroz, Andrea da Costa Moreira de Oliveira, Renato Carneiro de Freitas Chaves, Lucas Araújo de Borges Moura, Daniel Sousa César, Flávio Takaoka, Ary Serpa

**Affiliations:** 1 Hospital Israelita Albert Einstein, São Paulo, SP, Brazil.; 2 Irmandade da Santa Casa de Misericórdia de Santos, Santos, SP, Brazil.; 3 Department of Intensive Care Medicine, University Medical Centers, Amsterdam University, Amsterdam, Netherlands.

**Keywords:** Electronic health records, Data accuracy, Data collection, Anesthesia, Critical care

## Abstract

Data collection for clinical research can be difficult, and electronic health record systems can facilitate this process. The aim of this study was to describe and evaluate the secondary use of electronic health records in data collection for an observational clinical study. We used Cerner Millennium^®^, an electronic health record software, following these steps: (1) data crossing between the study’s case report forms and the electronic health record; (2) development of a manual collection method for data not recorded in Cerner Millennium^®^; (3) development of a study interface for automatic data collection in the electronic health records; (4) employee training; (5) data quality assessment; and (6) filling out the electronic case report form at the end of the study. Three case report forms were consolidated into the electronic case report form at the end of the study. Researchers performed daily qualitative and quantitative analyses of the data. Data were collected from 94 patients. In the first case report form, 76.5% of variables were obtained electronically, in the second, 95.5%, and in the third, 100%. The daily quality assessment of the whole process showed complete and correct data, widespread employee compliance and minimal interference in their practice. The secondary use of electronic health records is safe and effective, reduces manual labor, and provides data reliability. Anesthetic care and data collection may be done by the same professional.

## INTRODUCTION

Continuous digital data registration of patient charts by implementation of Electronic Health Record (EHR) software has enabled continuous data collection during patient care and production of large secondary databases. The need to intersect clinical research data with data collected and registered by EHR^1^ daily during patient management has stimulated the advent of innovative methods of clinical research data collection, employing the EHR as a major tool during the process.^2^

The anesthetic procedure requires nonstop delivery of patient care, making manual anesthesia registrations difficult, often resulting in incomplete and inaccurate data.^3^ Devices enabling automatic transfer of intraoperative monitoring data are available EHR resources, allowing accurate data acquisition, preventing clinical data losses and decreasing manual workload during this stage.^4^ Without EHR resources, collaboration with clinical research activities concurrent to anesthetic patient care delivery is challenging. In addition, the presence of staff with exclusive data collecting functions is expensive and impractical for most hospitals. Consequently, using EHR to assist clinical research has become an attractive approach, mostly in the intraoperative setting.

The primary objective of the present study was to describe the approach designed to integrate the EHR system to the case report form (CRF) of an intraoperative clinical study, enabling automatic data collection. The secondary objectives were to quantify the automatically collected data and assess their quality.

## METHODS

The study was performed in November 2017 at *Hospital Israelita Albert Einstein* (HIAE), in São Paulo (SP), Brazil.

### Study design Assessment of Ventilatory Management during General Anesthesia for Robotic Surgery and Its Effects on Postoperative Pulmonary Complications

**Figure 1:**
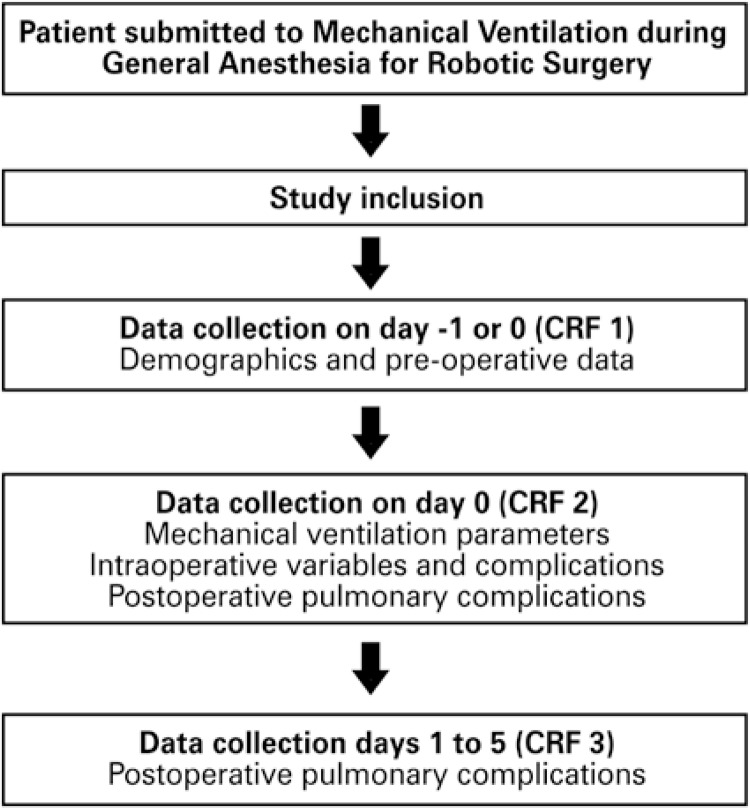
AVATaR (Assessment of Ventilatory Management During General Anesthesia for Robotic Surgery and Its Effects on Postoperative Pulmonary Complications) study flow

Assessment of Ventilatory Management During General Anesthesia for Robotic Surgery and Its Effects on Postoperative Pulmonary Complications *(*AVATaR)^5^ is a prospective, observational and multicenter study. The primary objective was to assess the incidence of post-operative pulmonary complications (PPC) in patients submitted to mechanical ventilation (MV) during robotic surgery. The secondary objective was to describe the current MV practice associated with patient positioning and the incidence of PPC (Figure 1). Presently the study is carried out at 29 centers, but the data collection methodology described herein refers only to data collected at HIAE.

CRF: case report form.

Moreover, HIAE is the study coordination center and the remaining centers are engaged in patient recruitment and local data collection. The centers included in the study are shown in table 1.

**Table 1:** Participating centers of the AVATaR study (Assessment of Ventilatory Management During General Anesthesia for Robotic Surgery and Its Effects on Postoperative Pulmonary Complications)

Center	City	Country
Bradford Teaching Hospitals NHS Foundation Trust	Bradford	United Kingdom
Cambridge University Hospitals NHS Foundation Trust	Cambridge	United Kingdom
Citta della Salute e della Scienza	Turin	Italy
City Hospitals Sunderland	Sunderland	United Kingdom
Consorcio Hospital General Universitario de Valencia	Valencia	Spain
Duesseldorf University Hospital	Dusseldorf	Germany
East Kent Hospitals University Foundation Trust	Kent	United Kingdom
East Lancashire Hospitals NHS Trust	Lancashire	United Kingdom
Fondazione Policlinico Universitario Gemelli of Rome	Rome	Italy
Hospital Clinic Barcelona	Barcelona	Spain
Hospital Clínico San Carlos	Madrid	Spain
*Hospital Israelita Albert Einstein*	São Paulo	Brazil
*Hospital Nove de Julho*	São Paulo	Brazil
Hull and East Yorkshire Hospitals NHS Trust site	Hull	United Kingdom
Kliniken Essen-Mitte	Essen	Germany
Maasstad Ziekenhuis	Rotterdam	Netherlands
Massachusetts General Hospital	Boston	United States
Mayo Clinic	Rochester	United States
MD Anderson Cancer Center	Houston	United States
Rabin Medical Center Beilinson Hospital	Petah Tikva	Israel
Royal Berkshire Hospital	Reading	United Kingdom
Royal Hallamshire	Sheffield	United Kingdom
Royal Surrey County Hospital NHS Foundation Trust	Guildford	United Kingdom
St George’s University Hospitals NHS Foundation Trust	Tooting	United Kingdom
Tel Aviv Medical Center	Tel Aviv	Israel
Università di Foggia	Foggia	Italy
University of California, San Francisco	San Francisco	United States
University of Genova	Genova	Italy
Wirral University Teaching Hospital NHS Foundation Trust	Wirral	United Kingdom

The study was approved by the Research Ethics Committee of HIAE (CAAE: 67113817.2.1001.007). The Informed Consent Form (ICF) was obtained from all patients included in the study. Finally, the tool described was used only to study eligible patients who signed the ICF. All collected data were encoded when entered in the final database, and their access password-protected and restricted to the principal investigator and the study’s statistician.

### AVATaR study data collection form

The final study CRF (Annex 1) was divided into three distinct forms, according to the perioperative period phases: (1) CRF-1, to collect preoperative data; (2) CRF-2, to collect intraoperative data; and (3) CRF-3, to collect postoperative data.

CRF-1 consisted of 77 items (14 major variables and 63 major variable subtypes) covering demographic data, baseline vital signs, physical and functional status, comorbidities, preoperative laboratory tests, previous respiratory complications, surgery type, expected duration of procedure and surgical incision site.

CRF-2 had two collection steps. The first stage included 52 items to fill out (21 major variables and 31 subtypes), describing details of anesthesia, surgical table positioning, carbon dioxide insufflation site, intraoperative fluid balance and duration of surgery and anesthesia. The second stage items described mechanical ventilation management during surgery collected at different moments: T_1_, 5 minutes after anesthesia induction and MV onset; T_2_, 5 minutes after the insufflation of carbon dioxide in the abdominal or thoracic cavity; T_3_, 5 minutes after the final surgical positioning; T_4_, every 60 minutes of intraoperative time (subdivided into T_4.1_, T_4.2_, T_4.3_ and so on up to the maximum number of 10 hours); and T_5_, 5 minutes after cavity deflation and final positioning at the end of surgery. The second stage of CRF-2 included 33 variables filled out from at least 5 moments to a maximum of 15 moments, depending on the surgical time, totaling from a minimum of 165 to a maximum of 495 variables.

Finally, CRF-3 had 21 variables related to patient recovery and the development of PPC, collected from day zero through day 5 or until hospital discharge, depending on what happened first.

### Electronic Health Record System

*Hospital Israelita Albert Einstein* implemented the EHR in January 2017, using the Cerner Millenniumt^®^ software. The surgical care flow was described on the Powerchart and SurgiNet *(*pre-anesthesia assessment and surgical reports) modules, and on the Saanesthesia (intraoperative anesthesia record).

**Figure 2:**
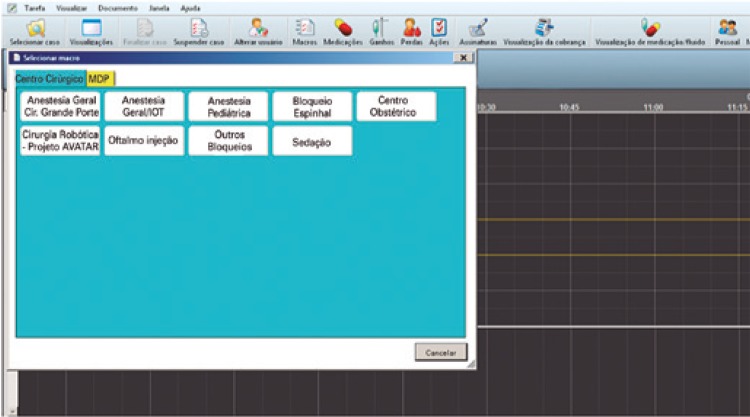
Initial screen of Projeto Macro Cirurgia Robótica - Assessment of Ventilatory Management During General Anesthesia for Robotic Surgery and Its Effects on Postoperative Pulmonary Complications (AVATaR)

Every 30 seconds, automatic migration of all parameters to the EHR occurs by integration between the EHR and the anesthesia machine, multi parameter monitor and bi-spectral index monitor, via the Intelligent Input Bus (IBus) method. Saanesthesia enabled the creation of an interface of the study, called *Macro Cirurgia Robótica Projeto* AVATaR [Macro Robotic Surgery AVATaR Project], by allowing the acquisition of data relevant to the study, which were not usually entered. Anesthesiologists enabled the tool at the beginning of the procedure (Figure 2).

ETI: endotracheal intubation.

It should be stressed that the EHR used for collecting study data is used routinely at HIAE for patient care after going through all required hospital safety procedures. Additionally, the EHR was validated previously to the beginning the study and complied with the norms of the ONC Health IT Certification Program. Also, the EHR does not allow data tampering; any data correction is performed by add-ons, and no information is deleted - just added. Finally, data contained in the EHR are part of the patient chart and abide to legal norms in force for such documentation. All pieces of information obtained through the EHR are recorded on the CRF on paper, which, together with the EHR, comprise a patient’s source document, as authorized by the research audit of the hospital. Paper documents are stored with the researchers responsible for the study.

### Secondary use of electronic support in data collection plan

**Figure 3:**
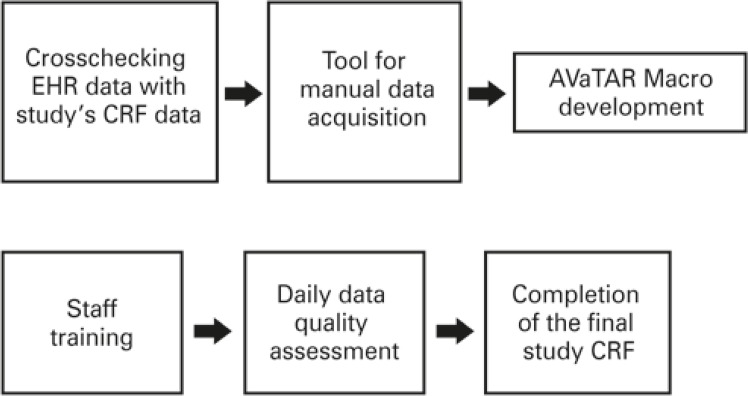
Development flow of the of the system for data collection

CRF: data collection form; AVATaR: Assessment of Ventilatory Management During General Anesthesia for Robotic Surgery and Its Effects on Postoperative Pulmonary Complications.

The final study CRF comprised three CRF, according to perioperative phases. Data collection was designed in six steps (Figure 3):

1. Crosschecking EHR routinely collected data with CRF.
2. Development at the Information Technology Department of the Macro AVATaR, for automatic intraoperative data acquisition (Figure 4).
  **Figure 4:**
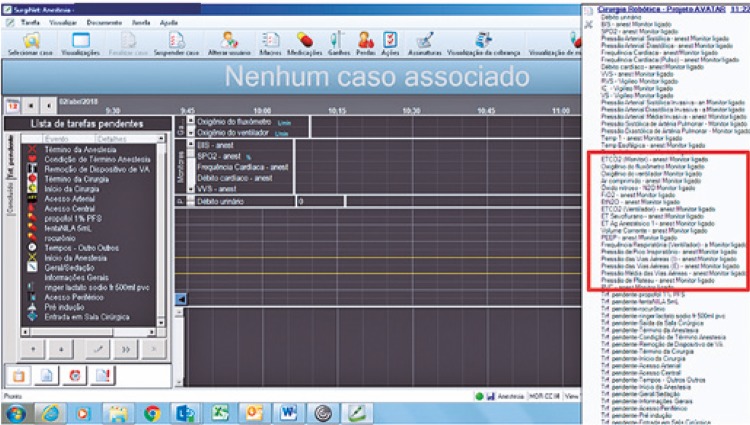
Data collection screen of the Macro Cirurgia Robótica Projeto - Assessment of Ventilatory Management During General Anesthesia for Robotic Surgery and Its Effects on Postoperative Pulmonary Complications (AVATaR)
3. Designing document for manual registering of CRF data not retrievable from EHR.
4. Training staff to obtain informed consent signature, to fill out manually collected data and to use the Macro AVATaR tool.
5. Daily data quality assessing by the principal investigator, by means of visiting patients and comprehensive review of EHR.
6. Filling out electronic CRF in the Research Electronic Data Capture (REDCap) system, by the principal investigator.

### Features of electronic data collection and of data management

All collected data were included in the electronic final CRF of the study on the REDCap^®^ through the internet. To ensure confidentiality, the form is hosted on HIAE servers in a secure and controlled environment. The system has the following functions: patient registering, data entry, data cleaning, audit trail and data export for statistical analysis.

All data entered undergo periodic management. The clinical data management plan provides high quality data by incorporating standardized procedures to minimize the number of errors and data loss, and thus generate a precise database for analysis. Remote monitoring is performed to flag aberrant patterns, problems with consistency, credibility, and other anomalies, according to predefined checking processes created in the system. Any missing and discrepant data values are reviewed individually and completed or corrected whenever possible.

## RESULTS

### Quantification of data collected

A total of 94 patients were included during the 30-day study period. Regarding CRF-1, from 34 variables, 26 (76.5%) were acquired electronically. For CRF-2, the value for electronic collected variables was 95.5%, or 214 out of 224 variables. As to CRF-3, all data (100%), of the 12 to 57 variables, depending on the length of hospital stay, were obtained electronically.

### Quality of data

For CRF-1, in 15 cases (15.6%), the principal investigator had to interview the patient for data completion. For three patients (3.1%), the “not previously smoker” condition was incorrectly entered as “former smoker” condition. During CRF-2 data acquisition, there was complete failure of automatic data migration for three patients, due to non-synchronization between monitoring devices and the Saanesthesia, due to misuse of the tool by the anesthetist. Only for five patients (5.2%) there was partial failure of data collection. No data correction was required after electronic data collection. No data correction was required in CRF-3 data collection.

## DISCUSSION

We described an approach for automatization of data collection for clinical research using EHR. We found variability in percentage rates of CRF completion using data obtained by the EHR. When comparing the forms collected, CRF-1 showed less data possible to be acquired electronically. The degree of intersections between the information required for completing the study CRF and the mandatory information present in the EHR explains the variation in performance. For instance, in CRF-3, all pertinent study data were already available on the HER, meanwhile in CRF-1, data were largely not available in the information routinely registered by the anesthesiologist, thus the data had to be acquired manually.

Employment of the EHR has a wide spectrum of applications in clinical research, ranging from secondary utilization of EHR supporting clinical research,^6^ to registry-based randomized controlled trials, which are entirely EHR-supported studies.^7^ Considering potential advantages, we can highlight cost reduction, fast patient recruitment, data produced in “real world” scenarios and the potential for a comprehensive follow up of patients.^7^ Ethics concerns are major challenges.^8^

Quality assessment of EHR-acquired data is challenging and the absence of devices ensuring the validation of EHR use as an assisting tool for research can be a source of bias.^9,10^ A previous prospective study assessed the potential use of EHR as a substitute method for collecting postoperative data from 358 patients and found that more than 96% of required data were completely filled out by the EHR, but the agreement rate among the data assessed showed variation ranging from 19 to 73%. Hence, the author stressed the need for assessing variable by variable for planning this data acquisition approach for clinical research.^11^

In many ways the solution was innovative, since it took advantage of the potentialities of an HER not primarily designed for clinical research, and enabled the anesthesiologist to collect data and, simultaneously, provide patient care, without compromising any of the two processes. The wide compliance by staff results from the minimal interference on anesthetic management triggered by data collection. Additionally, the tool has helped produce real and checkable data, that can avoid manipulations, frauds and the occurrence of publications leading to scientific retractions.^12^ In an environment where discredit in scientific papers` veracity is an object of publications,^13^ technology can be a major ally, although functionally active researchers cannot yet be dispensed, because they can identify and correct process failures.

## CONCLUSION

We described an efficient approach to using an Electronic Health Record secondary application for acquiring research data. The approach has enabled the acquisition of data for a major clinical study simultaneously to clinical care. The help in collecting data provided by the tool has the potential of minimizing manual workload and thus, increasing staff compliance, and improving collected data quality. Additionally, due to double checking of all variables, we found that the approach can provide reliable and high-quality data. The present study showed the great potential application of Electronic Health Record to assist collecting data for clinical studies when its use is carefully planned.
