# Supplementary material for: Methodological description of clinical research data collection through electronic medical records in a center participating in an international multicenter study
Source: Einstein (Sao Paulo). 2019 Sep 16;17(4):eAE4791. doi: 10.31744/einstein_journal/2019AE4791 (PMC6748344; doi:10.31744/einstein_journal/2019AE4791)
Supplement: Supplementary file 1 [file 2317-6385-eins-17-04-eAE4791-suppl01.pdf]

**Annex 1**

CONFIDENTIAL

## MECHANICAL VENTILATION DURING ROBOTIC SURGERY (AVATaR)

Version 1.5

**Assessment of ventilation management during general anesthesia for robotic surgery and its effects on postoperative pulmonary complications: a multicenter prospective observational study**

Patient Serial Number |_|_|_|_|_|_| 
center patient

Investigator Site 1 ________________________

Principal Investigator: Prof. Ary Serpa Neto, Intensive Care Department, *Hospital Israelita Albert Einstein*

Contact: Veronica Neves Fialho Queiroz, Anesthesiology, *Hospital Israelita Albert Einstein*, veronicanfialho@gmail.com

**Patient submitted to Mechanical Ventilation during General Anesthesia for Robotic Surgery**

↓

**Study inclusion**

↓

**Data collection on day -1 or 0 (CRF 1)**
Demographics and pre-operative data

↓

**Data collection on day 0 (CRF 2)**
Mechanical ventilation parameters
Intraoperative variables and complications
Postoperative pulmonary complications

↓

**Data collection days 1 to 5 (CRF 3)**
Postoperative pulmonary complications

### 1. INCLUSION, EXCLUSION CRITERIA AND INFORMED CONSENT

| | Yes | No |
|---|---|---|
| Inclusion criteria | | |
| Age >18 years | ☐ | ☐ |
| Mechanical ventilation for robotic surgery | ☐ | ☐ |
| Exclusion criteria | | |
| Procedure during pregnancy | ☐ | ☐ |
| Procedure performed outside the operating room | ☐ | ☐ |
| Included patient (all "yes" inclusion criteria and all "no" exclusion criteria) | ☐ | ☐ |
| Informed consent required | ☐ | ☐ |
| Date of signature of consent | ____ / ____ / 20__ | |

### 2. DETAILS OF PATIENT AND PROCEDURE

Demographic data

Age (years): _________ Gender: male ☐ female ☐

Height (cm): _________ Weight (kgs): _________

Race: Caucasian ☐ African ☐ Hispanic ☐ Asian ☐ other ☐ ASA: 1 ☐ 2 ☐ 3 ☐ 4 ☐ 5 ☐

Functional status:
Independent ☐ partially dependent ☐ totally dependent ☐

continue...

...Continuation

**Annex 1**

| 2. DETAILS OF PATIENT AND PROCEDURE | | | |
|---|---|---|---|
| Comorbidities | | | |
| Hypertension | yes ☐ no ☐ | | |
| Coronary disease | yes ☐ no ☐ | | |
| Atrial fibrillation/flutter | yes ☐ no ☐ | if yes | acute ☐ paroxistic ☐ chronic ☐ |
| Heart failure | yes ☐ no ☐ | if yes | NYHA score (1-4):_________ |
| *Diabetes mellitus* | yes ☐ no ☐ | if yes | diet ☐ oral drug ☐ insulin ☐ |
| COPD | yes ☐ no ☐ | if yes | inhalation therapy ☐ corticoid ☐ |
| Asthma | yes ☐ no ☐ | | |
| Respiratory infection <30 days | yes ☐ no ☐ | if yes | upper ☐ lower ☐ |
| Smoker | yes ☐ no ☐ | if yes | current ☐ previous (stopped >3 months) ☐ |
| Obstructive sleep apnea | yes ☐ no ☐ | | |
| Active cancer | yes ☐ no ☐ | if yes | Type: _________ |
| Liver cirrhosis | yes ☐ no ☐ | | |
| Use of MV <30 days | yes ☐ no ☐ | | |
| Transfusion of packed RBC <30 days | yes ☐ no ☐ | | |
| Anemia (Hb <10 g/dL) | yes ☐ no ☐ | | |
| Chronic kidney failure | yes ☐ no ☐ | if yes | hemodialysis ☐ conservative ☐ |
| Current organic function | | | |
| Respiratory rate (/min) | _________ | | |
| Heart rate (/min) | _________ | | |
| Mean blood pressure (mmHg) | _________ | | |
| $SpO_2$ in room air and supine (%) | _________ | | |
| Hemoglobin (if available) | mmol/L ☐ g/dL ☐ | | _________ |
| Leucocytes (if available) | $x10^9$ ☐ cel/$mm^3$ ☐ | | _________ |
| Creatinine (if available) | mmol/L ☐ mg/dL ☐ | | _________ |
| Characteristics of procedure and anesthesia | | | |
| Procedure | elective ☐ urgency ☐ emergency ☐ | | |
| Anticipated duration | ≤2 hours ☐ 2 a 3 hours ☐ >3 hours ☐ | | |
| Surgical incision | peripheral ☐ low abdominal ☐ high abdominal ☐ thoracic ☐ other ☐:_________ | | |
| Surgical procedure | prostatectomy ☐ nephrectomy ☐ hysterectomy ☐ bariatric ☐ sacrocolpopexy ☐ cholecystectomy ☐ heart surgery ☐ colorectal resection ☐ hernia repair ☐ head and neck ☐ pulmonary resection ☐ other ☐:_________ | | |

**3. DEFINITIONS**

Cm: centimeters
COPD: chronic obstructive pulmonary disease
RBC: red blood cells
$SpO_2$: pulse oximetry
Kgs: kilograms
MV: mechanical ventilation
Hb: hemoglobin

Functional status:
- Independent: does not require any help for daily activities
- Partially dependent: requires some help for daily activities
- Totally dependent: requires help for all daily activities

ASA (American Society of Anesthesiologist):
- 1: Healthy patient without organic changes
- 2: Patient with mild or moderate systemic changes
- 3: Patient with severe systemic changes with functional limitation
- 4: Patient with severe systemic changes that are life-threatening
- 5: Moribund patient not expected to survive without surgery

NYHA (New York Heart Association Functional Classification):
- 1: Heart condition without symptoms or limitations for daily activities (no dyspnea)
- 2: Mild symptoms and minor limitation for daily activities (exertion dyspnea)
- 3: Moderate symptoms and limitations for daily activities (minimum exertion dyspnea)
- 4: Severe symptoms and limitations for daily activities (rest dyspnea)

Procedure:
- Emergency: surgery performed when a patient`s life is in danger
- Urgency: surgery required within 48 hours
- Elective: previously scheduled surgery, since it is not a medical emergency

continue...

...Continuation

**Annex 1**

## 1. CHARACTERISTICS OF INTRAOPERATIVE PERIOD

| | | | |
|---|---|---|---|
| Characteristics of anesthesia and procedure | | | |
| Type of tracheal prosthesis | simple ☐ double-lumen ☐ nasotraqueal ☐ bronchus blocker ☐ endobronchial ☐ | | |
| Type of anesthesia | total intravenous ☐ inhalational ☐ balanced ☐ | | |
| Antibiotic prophylaxis | yes ☐ no ☐ | | |
| One lung ventilation | yes ☐ no ☐ | if yes | Ventilated lung: R ☐ L ☐ duration (min): ________ |
| Neuroaxis block | yes ☐ no ☐ | if yes | epidural ☐ spinal ☐ combined epidural spinal anesthesia ☐ |
| Neuromuscular monitoring | yes ☐ no ☐ | if yes | EMG ☐ MMG ☐ AMG ☐ |
| Trendelenburg | yes ☐ no ☐ | if yes | normal ☐ extreme (≥40°) ☐ |
| Conversion to open technique | yes ☐ no ☐ | | |
| Conversion to laparoscopy | yes ☐ no ☐ | | |
| $CO_2$ Insufflation | yes ☐ no ☐ | if yes | abdominal ☐ thoracic ☐ mediastinum ☐ |
| Duration of anesthesia (min): ________from intubation to extubation (or exit of operating room if on mechanical ventilation) | | | |
| Duration of surgery (min): ________from incision to closing | | | |
| Drugs, fluids and transfusion | | | |
| Crystalloid | yes ☐ no ☐ | if yes | Total volume (mL): ________ |
| Synthetic colloid | yes ☐ no ☐ | if yes | Total volume (mL): ________ |
| Albumin | yes ☐ no ☐ | if yes | Total volume (mL): ________ |
| Packed red blood cells | yes ☐ no ☐ | if yes | Total volume (un): ________ |
| Opioids | yes ☐ no ☐ | if yes | short duration ☐ long duration ☐ |
| Neuromuscular blocker | yes ☐ no ☐ | if yes | Rocuronium ☐ Vecuronium ☐ Atracurium ☐ Cisatracurium ☐ Pancuronium ☐ |
| Pharmacological reversal of neuromuscular block | yes ☐ no ☐ | if yes | Sugammadex ☐ Neostigmine ☐ Pyridostigmine ☐ Physostigmine ☐ |
| End of anesthesia | | | |
| Estimated blood loss | yes ☐ no ☐ | if yes | Total volume (mL): ________ |
| Urinary output | yes ☐ no ☐ | if yes | Total volume (mL): ________ |
| Residual curarization | yes ☐ no ☐ | | |
| Temperature | yes ☐ no ☐ | if yes | value (°C): ________ |
| Post-operative epidural | yes ☐ no ☐ | | |
| Complications during intraoperative period | | | |
| Desaturation ($SpO_2$ <92% for 3 minutes or more) | | | yes ☐ no ☐ |
| Recruitment maneuver not previously planned | | | yes ☐ no ☐ |
| Reduction in airway pressure | | | yes ☐ no ☐ |
| Hypotension (SAP <90mmHg or MAP <65mmHg for 3 minutes or more) | | | yes ☐ no ☐ |
| Acute arrythmia (AF, VT, SVT or VF) | | | yes ☐ no ☐ |
| Not anticipated vasoactive drug and on continuous infusion required | | | yes ☐ no ☐ |

## 2. DEFINITIONS

Ventilated lung: R (right) or L (left)

EMG: electromyography

MMG: mechanomyography

AMG: acceleromyography

mL: milliliters

UN: units

$SpO_2$: pulse oximetry

SAP: systolic arterial pressure

MAP: mean arterial pressure

FA: atrial fibrillation

VT: ventricular tachycardia

SVT: supraventricular tachycardia

VF: ventricular fibrillation

°C: degrees Celsius

Min: minutes

Residual curarization
Defined as train-of-four (TOF) stimulation rate <0.9 or diagnosed clinically

Opioids
Short duration: alfentanil, fentanyl, sufentanil, remifentanil, morphine
Long duration: opioids as extended or prolonged release formulation

Desaturation
Defined as $SpO_2$ <92% for 3 minutes or more

Hypotension
Defined as systolic arterial pressure <90mmHg or mean arterial pressure <65mmHg for 3 minutes or more

continue...

...Continuation

**Annex 1**

### 2. DEFINITIONS

Arrythmia

Atrial fibrillation (AF): defined for absolute irregularity of R-R intervals and simultaneous loss of P waves identifiable on EKG recordings

Sustained ventricular tachycardia (VT): characterized by ≥3 consecutive QRS complex with a wide QRS complex at a HR >100 beats/min and duration >30 seconds

Supraventricular tachycardia (SVT): identified as a narrow QRS complex (<0.12 seconds) and a HR >180 beats/min

Ventricular fibrillation (VF): defined as a chaotic electrical ventricular activity, with pronounced variability in QRS morphology, amplitude and cycle

Vasoactive drug required

Any non-anticipated and of continuous infusion vasoactive drug required. Drugs considered: phenylephrine, vasopressin, dopamine, norepinephrine, epinephrine, dobutamine, ephedrine, atropine and/or milrinone

### 3. VENTILATION PARMETERS

| | $T_1$ | $T_2$ | $T_3$ | $T_{4.1}$ | $T_{4.2}$ | $T_{4.3}$ | $T_{4.4}$ | $T_{4.5}$ | $T_{4.6}$ | $T_{4.7}$ | $T_{4.8}$ | $T_{4.9}$ | $T_{4.10}$ | $T_5$ |
|---|---|---|---|---|---|---|---|---|---|---|---|---|---|---|
| Ventilation parameters | | | | | | | | | | | | | | |
| Mode | PC ☐<br>VC ☐ | PC ☐<br>VC ☐ | PC ☐<br>VC ☐ | PC ☐<br>VC ☐ | PC ☐<br>VC ☐ | PC ☐<br>VC ☐ | PC ☐<br>VC ☐ | PC ☐<br>VC ☐ | PC ☐<br>VC ☐ | PC ☐<br>VC ☐ | PC ☐<br>VC ☐ | PC ☐<br>VC ☐ | PC ☐<br>VC ☐ | PC ☐<br>VC ☐ |
| Ppeak ($cmH_2O$) | | | | | | | | | | | | | | |
| Pplateau ($cmH_2O$) | | | | | | | | | | | | | | |
| Pmean ($cmH_2O$) | | | | | | | | | | | | | | |
| PEEP ($cmH_2O$) | | | | | | | | | | | | | | |
| $V_T$ inspired (mL) | | | | | | | | | | | | | | |
| RR (/min) | | | | | | | | | | | | | | |
| I:E | | | | | | | | | | | | | | |
| $FiO_2$ (%) | | | | | | | | | | | | | | |
| RA | PEEP ☐<br>$V_T$ ☐<br>Double ☐<br>Bag ☐<br>CPAP ☐<br>No ☐ | PEEP ☐<br>$V_T$ ☐<br>Double ☐<br>Bag ☐<br>CPAP ☐<br>No ☐ | PEEP ☐<br>$V_T$ ☐<br>Double ☐<br>Bag ☐<br>CPAP ☐<br>No ☐ | PEEP ☐<br>$V_T$ ☐<br>Double ☐<br>Bag ☐<br>CPAP ☐<br>No ☐ | PEEP ☐<br>$V_T$ ☐<br>Double ☐<br>Bag ☐<br>CPAP ☐<br>No ☐ | PEEP ☐<br>$V_T$ ☐<br>Double ☐<br>Bag ☐<br>CPAP ☐<br>No ☐ | PEEP ☐<br>$V_T$ ☐<br>Double ☐<br>Bag ☐<br>CPAP ☐<br>No ☐ | PEEP ☐<br>$V_T$ ☐<br>Double ☐<br>Bag ☐<br>CPAP ☐<br>No ☐ | PEEP ☐<br>$V_T$ ☐<br>Double ☐<br>Bag ☐<br>CPAP ☐<br>No ☐ | PEEP ☐<br>$V_T$ ☐<br>Double ☐<br>Bag ☐<br>CPAP ☐<br>No ☐ | PEEP ☐<br>$V_T$ ☐<br>Double ☐<br>Bag ☐<br>CPAP ☐<br>No ☐ | PEEP ☐<br>$V_T$ ☐<br>Double ☐<br>Bag ☐<br>CPAP ☐<br>No ☐ | PEEP ☐<br>$V_T$ ☐<br>Double ☐<br>Bag ☐<br>CPAP ☐<br>No ☐ | PEEP ☐<br>$V_T$ ☐<br>Double ☐<br>Bag ☐<br>CPAP ☐<br>No ☐ |
| Vital parameters | | | | | | | | | | | | | | |
| $SpO_2$ (%) | | | | | | | | | | | | | | |
| $etCO_2$ (mmHg) | | | | | | | | | | | | | | |
| MAP (mmHg) | | | | | | | | | | | | | | |
| HR (/min) | | | | | | | | | | | | | | |
| Others | | | | | | | | | | | | | | |
| Ppneumo (mmHg) | | | | | | | | | | | | | | |
| Highest value at time | | | | | | | | | | | | | | |
| Position | HD ☐<br>HV ☐<br>LD ☐<br>LIT ☐<br>T ☐<br>RT ☐<br>S ☐ | HD ☐<br>HV ☐<br>LD ☐<br>LIT ☐<br>T ☐<br>RT ☐<br>S ☐ | HD ☐<br>HV ☐<br>LD ☐<br>LIT ☐<br>T ☐<br>RT ☐<br>S ☐ | HD ☐<br>HV ☐<br>LD ☐<br>LIT ☐<br>T ☐<br>RT ☐<br>S ☐ | HD ☐<br>HV ☐<br>LD ☐<br>LIT ☐<br>T ☐<br>RT ☐<br>S ☐ | HD ☐<br>HV ☐<br>LD ☐<br>LIT ☐<br>T ☐<br>RT ☐<br>S ☐ | HD ☐<br>HV ☐<br>LD ☐<br>LIT ☐<br>T ☐<br>RT ☐<br>S ☐ | HD ☐<br>HV ☐<br>LD ☐<br>LIT ☐<br>T ☐<br>RT ☐<br>S ☐ | HD ☐<br>HV ☐<br>LD ☐<br>LIT ☐<br>T ☐<br>RT ☐<br>S ☐ | HD ☐<br>HV ☐<br>LD ☐<br>LIT ☐<br>T ☐<br>RT ☐<br>S ☐ | HD ☐<br>HV ☐<br>LD ☐<br>LIT ☐<br>T ☐<br>RT ☐<br>S ☐ | HD ☐<br>HV ☐<br>LD ☐<br>LIT ☐<br>T ☐<br>RT ☐<br>S ☐ | HD ☐<br>HV ☐<br>LD ☐<br>LIT ☐<br>T ☐<br>RT ☐<br>S ☐ | HD ☐<br>HV ☐<br>LD ☐<br>LIT ☐<br>T ☐<br>RT ☐<br>S ☐ |

### 4. DEFINITIONS

Ppeak: peak pressure

Pmean: mean pressure

$V_T$: tidal volume

I:E: inspiration:expiration ratio

$etCO_2$: end of expiration $CO_2$ exhaled

HR: Heart rate

Plateau: plateau pressure

Plateau pressure should be measured using an inspiratory pause of at least 5 seconds

PEEP: positive end expiration pressure

RR: respiratory rate

$FiO_2$: inspired oxygen fraction

$SpO_2$: pulse oximetry

MAP: mean arterial pressure

Ppneumo: pneumoperitoneum pressure

RA: alveolar recruitment maneuver

PEEP: gradual increase in PEEP with constant tidal volume

$V_T$: gradual increase in volume with constant PEEP

Double: PEEP and tidal volume are both gradually increased

Bag: manual hyperinflation with balloon/bag

CPAP: positive pressure on airways over 30$cmH_2O$ applied during 10 to 30 seconds

continue...

...Continuation

**Annex 1**

## 4. DEFINITIONS

Mode: ventilatory mode
CP: controlled pressure
$V_T$: tidal volume

Position: position during surgical procedure
HDD: horizontal dorsal decubitus (supine)
HVD: horizontal ventral decbitus (prone)
LD: lateral decubitus
LIT: lithotomy
T: Trendelenburg
RT: Reverse Trendelenburg
S: seated

T: surgical moments
1: 5 minutes after initiating mechanical ventilation
2: 5 minutes after performing pneumoperitoneum (do not fill out if not performed)
3: 5 minutes after definitive intraoperative positioning
4.1: 60 minutes after T3
4.2-4.10: every 60 minutes
5: 5 minutes after pneumoperitoneum evacuation (if performed) and final positioning

## 1. POSTOPERATIVE VISIT ON DAY 0 (END OF SURGERY UNTIL 11:59 pm)

| | | | |
|---|---|---|---|
| Recovery | | | |
| Loss to follow-up | yes ☐ no ☐ | if yes | discharge ☐ death ☐ transfer ☐ other ☐: _________ |
| Continuation of MV after surgery | yes ☐ no ☐ | if yes | planned ☐ not planned (continuous) ☐ not planned (reintubation) ☐ |
| If reintubation, cause | ARF ☐ ALOC ☐ hemodynamic instability ☐ | | |
| Admission to ICU after surgery | yes ☐ no ☐ | if yes | planned ☐ not planned ☐ |
| Postoperative pulmonary complications | | | |
| Oxygen required<br>$PaO_2$ <60mmHg or $SpO_2$ <90% in RA | yes ☐ no ☐ | if yes | $FiO_2$ (%) offered: _________ |
| Acute respiratory failure<br>$PaO_2$ <60mmHg or $SpO_2$ <90% with oxygen or NIV | yes ☐ no ☐ | if yes | NIV: yes ☐ no ☐ if yes,<br>Interface: mask ☐ helmet ☐ |
| Pneumonia<br>new/worsening infiltrated + 2: fever, leukocytosis/leucopenia, purulent discharge, antibiotic | yes ☐ no ☐ | | |
| ARDS<br>According to Berlin criteria | yes ☐ no ☐ | if yes | mild ☐ moderate ☐ severe ☐ |
| Pneumothorax<br>air between visceral and parietal pleura | yes ☐ no ☐ | | |

## 2. DEFINITIONS

MV: mechanical ventilation
$FiO_2$: inspired oxygen fraction
$PaO_2$: partial oxygen pressure
RA: room air
ARF: acute respiratory failure

ICU: intensive care unit
NIV: non-invasive ventilation
$SpO_2$: pulse oximetry
ARDS: acute respiratory discomfort syndrome
ALOC: altered level of consciousness

Oxygen required
Defined as supplementary oxygen used due to $PaO_2$ <60mmHg or $SpO_2$ <92% in room air (in individuals without previous lung disease) or $SpO_2$ <88% (in individuals with previous lung disease)

Acute respiratory failure
Defined as $PaO_2$ <60mmHg or $SpO_2$ <92%, despite oxygen therapy, or non-invasive (NIV) mechanical ventilation required

Pneumonia
Defined by the presence of new or progressive radiographic infiltrate, in addition to at least two to four clinical characteristics: fever >38°C, leukocytosis or leucopenia (leucocyte count >12,000 cells/mm or <4,000 cells/mm$^3$), purulent discharge or use of antibiotic

Acute Respiratory Discomfort Syndrome (ARDS)
Time: within a week of a known clinical insult or worsening of respiratory symptoms
Image: bilateral opacities not totally explained by pleural effusions, pulmonary or lobar collapse or nodules (chest x-ray or computerized tomography)
Origin of edema: Respiratory failure not totally explained by heart failure or volume overload. Objective assessment required (example: echocardiography) to exclude hydrostatic edema if there is no risk factor present
Oxygenation:
Mild: 200mmHg$<PaO_2/FiO_2 \leq$300mmHg with PEEP or CPAP $\geq$5cm$H_2O$ (can be by NIV)
Moderate: 100mmHg$<PaO_2/FiO_2\leq$200mmHg with PEEP
Severe: 100mmHg$\leq PaO_2/FiO_2$ with PEEP

Pneumothorax
Defined as presence of air between visceral and parietal pleura, the diagnosis can be made by physical examination and chest x-ray

continue...

...Continuation

**Annex 1**

### 1. POSTOPERATIVE VISIT DAY 1 (FROM 0:00 am TO 11:59 pm)

Recovery

| | | | |
|---|---|---|---|
| Loss to follow-up | yes ☐ no ☐ | if yes | discharge ☐ death ☐ transfer ☐ other ☐: ________ |
| New MV required | yes ☐ no ☐ | | |
| Admission to ICU required | yes ☐ no ☐ | | |

Postoperative pulmonary complications

| | | | |
|---|---|---|---|
| Oxygen required<br>$PaO_2$ <60mmHg or $SpO_2$ <90% in RA | yes ☐ no ☐ | if yes | $FiO_2$ (%) offered: ________ |
| Acute respiratory failure<br>$PaO_2$ <60mmHg or $SpO_2$ <90% with oxygen or NIV required | yes ☐ no ☐ | if yes | NIV: yes ☐ no ☐ if yes,<br>Interface: mask ☐ helmet ☐ |
| Pneumonia<br>new/worsening infiltrate + 2: fever, leukocytosis/leucopenia, purulent discharge, antibiotic | yes ☐ no ☐ | | |
| ARDS<br>according to Berlin criteria | yes ☐ no ☐ | if yes | mild ☐ moderate ☐ severe ☐ |
| Pneumothorax<br>air between visceral and parietal pleura | yes ☐ no ☐ | | |

### 2. DEFINITION

MV: mechanical ventilation

ICU: intensive care unit

$FiO_2$: inspired oxygen fraction

NIV: non-invasive ventilation

$PaO_2$: partial oxygen pressure

$SpO_2$: pulse oximetry

RA: room air

ARDS: acute respiratory discomfort syndrome

Oxygen required

defined as supplementary oxygen used due to $PaO_2$ <60mmHg or $SpO_2$ <92% in room air (in individuals without previous pulmonary disease) or $SpO_2$ <88% (in individuals with previous pulmonary disease)

Acute respiratory failure

defined as $PaO_2$ <60mmHg or $SpO_2$ <92%, despite oxygen therapy, or required non-invasive mechanical ventilation (NIV)

Pneumonia

defined by the presence of new or progressive radiographic infiltrate, in addition to at least two of four clinical characteristics: fever >38°C, leukocytosis or leucopenia (leucocyte count >12,000 cells/mm or <4,000 cells/mm$^3$), purulent discharge or use of antibiotic

Acute Respiratory Discomfort Syndrome (ARDS)

Time: within one week of known clinical insult or worsening of respiratory symptoms

Image: bilateral opacities not totally explained by pleural effusions, pulmonary or lobar collapse or nodules (chest x-ray or computerized tomography)

Origin of edema: respiratory failure not totally explained by heart failure or volume overload. Needs objective assessment (example: echocardiography) to exclude hydrostatic edema, if there is no risk factor present

Oxygenation:

Mild: 200mmHg < $PaO_2/FiO_2$ ≤300mmHg with PEEP or CPAP ≥5cm$H_2O$ (can be via NIV)

Moderate: 100mmHg < $PaO_2/FiO_2$ ≤200mmHg with PEEP

Severe: 100mmHg ≤ $PaO_2/FiO_2$ with PEEP

Pneumothorax

defined as the presence of air between visceral and parietal pleura, the diagnosis can be made by physical examination and chest x-ray

### 1. POSTOPERATIVE VISIT ON DAY 2 (FROM 0:00 am TO 11:59 pm)

Recovery

| | | | |
|---|---|---|---|
| Loss to follow-up | yes ☐ no ☐ | if yes | discharge ☐ death ☐ transfer ☐ other ☐: ________ |
| New MV required | yes ☐ no ☐ | | |
| Admission to ICU required | yes ☐ no ☐ | | |

Postoperative pulmonary complications

| | | | |
|---|---|---|---|
| Oxygen required<br>$PaO_2$ <60mmHg or $SpO_2$ <90% in RA | yes ☐ no ☐ | if yes | $FiO_2$ (%) offered: ________ |
| Acute respiratory failure<br>$PaO_2$ <60mmHg or $SpO_2$ <90% with oxygen or NIV required | yes ☐ no ☐ | if yes | Use of NIV: yes ☐ no ☐ if yes<br>Interface: mask ☐ helmet ☐ |
| Pneumonia<br>new/worsening infiltrate + 2: fever, leukocytosis/leucopenia, purulent discharge, antibiotic | yes ☐ no ☐ | | |
| ARDS<br>according to Berlin criteria | yes ☐ no ☐ | if yes | mild ☐ moderate ☐ severe ☐ |
| Pneumothorax<br>air between visceral and parietal pleura | yes ☐ no ☐ | | |

continue...

...Continuation

**Annex 1**

## 2. DEFINITIONS

| | |
|---|---|
| MV: mechanical ventilation | ICU: intensive care unit |
| $FiO_2$: inspired oxygen fraction | NIV: non-invasive ventilation |
| $PaO_2$: partial oxygen pressure | $SpO_2$: pulse oximetry |
| RA: room air | ARDS: acute respiratory discomfort syndrome |

Oxygen required
defined as supplementary oxygen used due to $PaO_2$ <60mmHg or $SpO_2$ <92% in room air (in individuals without previous pulmonary disease) or $SpO_2$ <88% (in individuals with previous pulmonary disease)

Acute respiratory failure
defined as $PaO_2$ <60mmHg or $SpO_2$ <92%, despite oxygen therapy, or non-invasive mechanical ventilation required (NIV)

Pneumonia
defined by the presence of new or progressive radiographic infiltrate, in addition to at least two of four clinical characteristics: fever >38°C, leukocytosis or leucopenia (leucocyte count >12,000 cells/mm or <4,000 cells/$mm^3$), purulent discharge or use of antibiotic

Acute Respiratory Discomfort Syndrome (ARDS)
Time: within one week of known clinical insult or worsening of respiratory symptoms
Image: bilateral opacities not totally explained by pleural effusions, pulmonary or lobar collapse or nodules (chest x-ray or computerized tomography)
Origin of edema: respiratory failure not totally explained by heart failure or volume overload. Needs objective assessment (example: echocardiography) to exclude hydrostatic edema, if there is no risk factor present
Oxygenation:
Mild: 200mmHg $<PaO_2/FiO_2 \leq$300mmHg with PEEP or CPAP ≥5cm$H_2O$ (can be via NIV)
Moderate: 100mmHg $<PaO_2/FiO_2 \leq$200mmHg with PEEP
Severe: 100mmHg $\leq PaO_2/FiO_2$ with PEEP

Pneumothorax
defined as the presence of air between visceral and parietal pleura, the diagnosis can be made by physical examination and chest x-ray

## 1. POSTOPERATIVE VISIT ON DAY 3 (FROM 0:00 am TO 11:59 pm)

| | | | |
|---|---|---|---|
| **Recovery** | | | |
| Loss to follow-up | yes ☐ no ☐ | if yes | discharge ☐ death ☐ transfer ☐ other ☐: ________ |
| New MV required | yes ☐ no ☐ | | |
| Admission to ICU required | yes ☐ no ☐ | | |
| **Postoperative pulmonary complications** | | | |
| Oxygen required<br>$PaO_2$ <60mmHg or $SpO_2$ <90% in RA | yes ☐ no ☐ | if yes | $FiO_2$ (%) offered: ________ |
| Acute respiratory failure<br>$PaO_2$ <60mmHg or $SpO_2$ <90% with oxygen or NIV required | yes ☐ no ☐ | if yes | Use of NIV: yes ☐ no ☐ if yes<br>Interface: mask ☐ helmet ☐ |
| Pneumonia<br>new/worsening infiltrate + 2: fever, leukocytosis/leucopenia, purulent discharge, antibiotic | yes ☐ no ☐ | | |
| ARDS<br>according to Berlin criteria | yes ☐ no ☐ | if yes | mild ☐ moderate ☐ severe ☐ |
| Pneumothorax<br>air between visceral and parietal pleura | yes ☐ no ☐ | | |

## 2. DEFINITIONS

| | |
|---|---|
| MV: mechanical ventilation | ICU: intensive care unit |
| $FiO_2$: inspired oxygen fraction | NIV: non-invasive ventilation |
| $PaO_2$: partial oxygen pressure | $SpO_2$: pulse oximetry |
| RA: room air | ARDS: acute respiratory discomfort syndrome |

Oxygen required
defined as supplementary oxygen used due to $PaO_2$ <60mmHg or $SpO_2$ <92% in room air (in individuals without previous pulmonary disease) or $SpO_2$ <88% (in individuals with previous pulmonary disease)

Acute respiratory failure
defined as $PaO_2$ <60mmHg or $SpO_2$ <92%, despite oxygen therapy, or non-invasive mechanical ventilation required (NIV)

Pneumonia
defined by the presence of new or progressive radiographic infiltrate, in addition to at least two of four clinical characteristics: fever >38°C, leukocytosis or leucopenia (leucocyte count >12,000 cells/mm or <4,000 cells/$mm^3$), purulent discharge or use of antibiotic

continue...

...Continuation

**Annex 1**

## 2. DEFINITIONS

Acute Respiratory Discomfort Syndrome (ARDS)
- Time: within one week of known clinical insult or worsening of respiratory symptoms
- Image: bilateral opacities not totally explained by pleural effusions, pulmonary or lobar collapse or nodules (chest x-ray or computerized tomography)
- Origin of edema: respiratory failure not totally explained by heart failure or volume overload. Needs objective assessment (example: echocardiography) to exclude hydrostatic edema, if there is no risk factor present

Oxygenation:
- Mild: 200mmHg $<PaO_2/FiO_2 \leq 300$mmHg with PEEP or CPAP $\geq 5cmH_2O$ (can be via NIV)
- Moderate: 100mmHg $<PaO_2/FiO_2 \leq 200$mmHg with PEEP
- Severe: 100mmHg $\leq PaO_2/FiO_2$ with PEEP

Pneumothorax
- defined as the presence of air between visceral and parietal pleura, the diagnosis can be made by physical

## 1. POSTOPERATIVE VISIT ON DAY 4 (FROM 0:00 am TO 11:59 pm)

| | | | |
|---|---|---|---|
| **Recovery** | | | |
| Loss to follow-up | yes ☐ no ☐ | if yes | discharge ☐ death ☐ transfer ☐ other ☐: ________ |
| New MV required | yes ☐ no ☐ | | |
| Admission to ICU required | yes ☐ no ☐ | | |
| **Postoperative pulmonary complications** | | | |
| Oxygen required<br>$PaO_2$ <60mmHg or $SpO_2$ <90% in RA | yes ☐ no ☐ | if yes | $FiO_2$ (%) offered: ________ |
| Acute respiratory failure<br>$PaO_2$ <60mmHg or $SpO_2$ <90% with oxygen or NIV required | yes ☐ no ☐ | if yes | Use of NIV: yes ☐ no ☐ if yes<br>Interface: mask ☐ helmet ☐ |
| Pneumonia<br>new/worsening infiltrate + 2: fever, leukocytosis/leucopenia, purulent discharge, antibiotic | yes ☐ no ☐ | | |
| ARDS<br>according to Berlin criteria | yes ☐ no ☐ | if yes | mild ☐ moderate ☐ severe ☐ |
| Pneumothorax<br>air between visceral and parietal pleura | yes ☐ no ☐ | | |

## 2. DEFINITIONS

| | |
|---|---|
| MV: mechanical ventilation | ICU: intensive care unit |
| $FiO_2$: inspired oxygen fraction | NIV: non-invasive ventilation |
| $PaO_2$: partial oxygen pressure | $SpO_2$: pulse oximetry |
| RA: room air | ARDS: acute respiratory discomfort syndrome |

Oxygen required
- defined as supplementary oxygen used due to $PaO_2$ <60mmHg or $SpO_2$ <92% in room air (in individuals without previous pulmonary disease) or $SpO_2$ <88% (in individuals with previous pulmonary disease)

Acute respiratory failure
- defined as $PaO_2$ <60mmHg or $SpO_2$ <92%, despite oxygen therapy, or non-invasive mechanical ventilation required (NIV)

Pneumonia
- defined by the presence of new or progressive radiographic infiltrate, in addition to at least two of four clinical characteristics: fever >38°C, leukocytosis or leucopenia (leucocyte count >12,000 cells/mm or <4,000 cells/mm$^3$), purulent discharge or use of antibiotic

Acute Respiratory Discomfort Syndrome (ARDS)
- Time: within one week of known clinical insult or worsening of respiratory symptoms
- Image: bilateral opacities not totally explained by pleural effusions, pulmonary or lobar collapse or nodules (chest x-ray or computerized tomography)
- Origin of edema: respiratory failure not totally explained by heart failure or volume overload. Needs objective assessment (example: echocardiography) to exclude hydrostatic edema, if there is no risk factor present

Oxygenation:
- Mild: 200mmHg $<PaO_2/FiO_2 \leq 300$mmHg with PEEP or CPAP $\geq 5cmH_2O$ (can be via NIV)
- Moderate: 100mmHg $<PaO_2/FiO_2 \leq 200$mmHg with PEEP
- Severe: 100mmHg $\leq PaO_2/FiO_2$ with PEEP

Pneumothorax
- defined as the presence of air between visceral and parietal pleura, the diagnosis can be made by physical examination and chest x-ray

continue...

...Continuation

**Annex 1**

| 1. POSTOPERATIVE VISIT ON DAY 5 (FROM 0:00 am TO 11:59 pm) | | | |
|---|---|---|---|
| Recovery | | | |
| Loss to follow-up | yes ☐ no ☐ | if yes | discharge ☐ death ☐ transfer ☐ other ☐: ________ |
| New MV required | yes ☐ no ☐ | | |
| Admission to ICU required | yes ☐ no ☐ | | |
| Postoperative pulmonary complications | | | |
| Oxygen required<br>$PaO_2$ <60mmHg or $SpO_2$ <90% in RA | yes ☐ no ☐ | if yes | $FiO_2$ (%) offered: ________ |
| Acute respiratory failure<br>$PaO_2$ <60mmHg or $SpO_2$ <90% with oxygen or NIV required | yes ☐ no ☐ | if yes | Use of NIV: yes ☐ no ☐ if yes<br>Interface: mask ☐ helmet ☐ |
| Pneumonia<br>new/worsening infiltrate + 2: fever, leukocytosis/leucopenia, purulent discharge, antibiotic | yes ☐ no ☐ | | |
| ARDS<br>according to Berlin criteria | yes ☐ no ☐ | if yes | mild ☐ moderate ☐ severe ☐ |
| Pneumothorax<br>air between visceral and parietal pleura | yes ☐ no ☐ | | |

**2. DEFINITIONS**

MV: mechanical ventilation

$FiO_2$: inspired oxygen fraction

$PaO_2$: partial oxygen pressure

RA: room air

ICU: intensive care unit

NIV: non-invasive ventilation

$SpO_2$: pulse oximetry

ARDS: acute respiratory discomfort syndrome

Oxygen required

defined as supplementary oxygen used due to $PaO_2$ <60mmHg or $SpO_2$ <92% in room air (in individuals without previous pulmonary disease) or $SpO_2$ <88% (in individuals with previous pulmonary disease)

Acute respiratory failure

defined as $PaO_2$ <60mmHg or $SpO_2$ <92%, despite oxygen therapy, or non-invasive mechanical ventilation required (NIV)

Pneumonia

defined by the presence of new or progressive radiographic infiltrate, in addition to at least two of four clinical characteristics: fever >38°C, leukocytosis or leucopenia (leucocyte count >12,000 cells/mm or <4,000 cells/$mm^3$), purulent discharge or use of antibiotic

Acute Respiratory Discomfort Syndrome (ARDS)

Time: within one week of known clinical insult or worsening of respiratory symptoms

Image: bilateral opacities not totally explained by pleural effusions, pulmonary or lobar collapse or nodules (chest x-ray or computerized tomography)

Origin of edema: respiratory failure not totally explained by heart failure or volume overload. Needs objective assessment (example: echocardiography) to exclude hydrostatic edema, if there is no risk factor present

Oxygenation:

Mild: 200mmHg $<PaO_2/FiO_2 \leq$300mmHg with PEEP or CPAP ≥5cm$H_2O$ (can be via NIV)

Moderate: 100mmHg $<PaO_2/FiO_2 \leq$200mmHg with PEEP

Severe: 100mmHg $\leq PaO_2/FiO_2$ with PEEP

Pneumothorax

defined as the presence of air between visceral and parietal pleura, the diagnosis can be made by physical examination and chest x-ray

**1. DISCHARGE VISIT (DAY OF DISCHARGE)**

Outcomes

Date of admission: ___ / ___ / _____ Date of discharge: ___ / ___ / _____

Length of hospital stay: _______ days

Death during hospital stay yes ☐ no ☐ if yes Date of death: ___ / ___ / _____

Comments:
