# Supplementary material for: Methodological description of clinical research data collection through electronic medical records in a center participating in an international multicenter study
Source: Einstein (Sao Paulo). 2019 Sep 16;17(4):eAE4791. doi: 10.31744/einstein_journal/2019AE4791 (PMC6748344; doi:10.31744/einstein_journal/2019AE4791)
Supplement: Supplementary file 2 [file 2317-6385-eins-17-04-eAE4791-suppl01-pt.pdf]

Anexo 1

CONFIDENCIAL

VENTILAÇÃO MECÂNICA DURANTE CIRÚRGIA ROBÓTICA (AVATaR)

Versão 1.5

Avaliação do manejo ventilatório durante anestesia geral para cirurgia robótica e seus efeitos em complicações pulmonares no pós-operatório: um estudo observacional prospectivo multicêntrico

Número Serial do Paciente |__|__|__|__|__|__|

centro paciente

Investigador Local 1 ______________________

Investigador Principal: Prof. Ary Serpa Neto, Departamento de Terapia Intensiva, Hospital Israelita Albert Einstein

Contato: Veronica Neves Fialho Queiroz, Anestesiologia, Hospital Israelita Albert Einstein, veronicanfialho@gmail.com

Paciente submetido a Ventilação Mecânica durante Anestesia Geral para Cirurgia Robótica

↓

Inclusão no estudo

↓

Aquisição dos dados no dia -1 ou 0 (CRF 1)
Demográficos e pré-operatórios

↓

Aquisição dos dados no dia 0 (CRF 2)
Parâmetros de ventilação mecânica
Variáveis e complicações intra-operatórias
Complicações pulmonares no pós-operatório

↓

Aquisição dos dados no dias 1 a 5 (CRF 3)
Complicações pulmonares no pós-operatórios

1. CRITÉRIOS DE INCLUSÃO, EXCLUSÃO E CONSENTIMENTO

	Sim	Não
Critérios de inclusão		
Idade >18 anos	☐	☐
Ventilação mecânica para cirurgia robótica	☐	☐
Critérios de exclusão		
Procedimento durante gestação	☐	☐
Procedimento realizado fora do centro cirúrgico	☐	☐
Paciente incluído no estudo (todos critérios de inclusão “sim” e todos critérios de exclusão “não”	☐	☐
Consentimento informado necessário	☐	☐
Data de assinatura do consentimento	____ / ____ / 20___	

2. DETALHES DO PACIENTE E PROCEDIMENTO

Dados demográficos

Idade (anos): ________ Genêro: homem ☐ mulher ☐

Altura (cm): ________ Peso (kgs): ________

Raça: caucasiano ☐ negro ☐ hispânico ☐ asiático ☐ outro ☐ ASA: 1 ☐ 2 ☐ 3 ☐ 4 ☐ 5 ☐

Status funcional:
Independente ☐ parcialmente dependente ☐ totalmente dependente ☐

continua...

...Continuação

Anexo 1

2. DETALHES DO PACIENTE E PROCEDIMENTO

Comorbidades			
Hipertensão	sim ☐ não ☐		
Doença coronariana	sim ☐ não ☐		
Fibrilação/*flutter* atrial	sim ☐ não ☐	se sim	aguda ☐ paroxística ☐ crônica ☐
Insuficiência cardíaca	sim ☐ não ☐	se sim	NYHA escore (1-4): _________
Diabetes mellitus	sim ☐ não ☐	se sim	dieta ☐ med. oral ☐ insulina ☐
DPOC	sim ☐ não ☐	se sim	terapia inalatória ☐ corticoide ☐
Asma	sim ☐ não ☐		
Infecção respiratória <30 dias	sim ☐ não ☐	se sim	alta ☐ baixa☐
Tabagismo	sim ☐ não ☐	se sim	atual ☐ prévio (parou >3 meses) ☐
Apneia obstrutiva do sono	sim ☐ não ☐		
Câncer ativo	sim ☐ não ☐	se sim	Tipo: _________
Cirrose hepática	sim ☐ não ☐		
Uso de VM <30 dias	sim ☐ não ☐		
Transfusão de CH <30 dias	sim ☐ não ☐		
Anemia (Hb <10 g/dL)	sim ☐ não ☐		
Insuficiência renal crônica	sim ☐ não ☐	se sim	hemodiálise ☐ conservador ☐
Função orgânica atual			
Frequência respiratória (/min)	_________		
Frequência cardíaca (/min)	_________		
Pressão arterial média (mmHg)	_________		
SpO_2 em ar ambiente e supino (%)	_________		
Hemoglobina (se disponível)	mmol/L ☐ g/dL ☐		_________
Leucócitos (se disponível)	$x10^9$ ☐ cel/mm^3 ☐		_________
Creatinina (se disponível)	mmol/L ☐ mg/dL ☐		_________
Características do procedimento e anestesia			
Condição do procedimento	eletivo ☐ urgência ☐ emergência ☐		
Duração prevista	≤2 horas ☐ 2 a 3 horas ☐ >3 horas ☐		
Incisão cirúrgica	periférica ☐ abdominal baixa ☐ abdominal alta ☐ torácica ☐ outra ☐:_________		
Procedimento cirúrgico	prostatectomia ☐ nefrectomia ☐ histerectomia ☐ bariátrica ☐ sacrocolpopexia ☐ colecistectomia ☐ cirurgia cardíaca ☐ colorretal ☐ reparo de hérnia ☐ cabeça e pescoço ☐ ressecção pulmonar ☐ outro ☐:_________		

3. DEFINIÇÕES

Cm: centímetros

DPOC: doença pulmonar obstrutiva crônica

CH: concentrado de hemácias

SpO_2: oximetria de pulso

Kgs: Quilogramas

VM: ventilação mecânica

Hb: hemoglobina

Status funcional:
- Independente: não necessita de qualquer auxílio para atividades diárias
- Parcialmente dependente: necessita de algum auxílio para atividades diárias
- Totalmente dependente: necessita de total auxílio para atividades diárias

ASA (*American Society of Anesthesiologist*):
- 1: Paciente sadio sem alterações orgânicas
- 2: Paciente com alteração sistêmica leve ou moderada
- 3: Paciente com alteração sistêmica grave com limitação funcional
- 4: Paciente com alteração sistêmica grave que representa risco de vida
- 5: Paciente moribundo que não é esperado sobreviver sem cirurgia

NYHA (*New York Heart Association Functional Classification*):
- 1: Doença cardíaca sem sintomas ou limitações em atividades cotidianas (sem dispneia)
- 2: Sintomas leves e limitação pequena para atividades cotidianas (dispneia aos esforços)
- 3: Sintomas e limitações moderadas para atividades cotidianas (dispneia aos mínimos esforços)
- 4: Sintomas e limitações graves para atividades cotidianas (dispneia em repouso)

Condição do procedimento:
- Emergência: cirurgia realizada quando a vida do paciente está em perigo
- Urgência: cirurgia necessária em 48 horas
- Eletiva: cirurgia que é programada com antecedência, pois não se trata de uma emergência médica

continua...

...Continuação

Anexo 1

1. CARACTERÍSTICAS DO INTRAOPERATÓRIO			
Características da anestesia e do procedimento			
Tipo de prótese traqueal	simples ☐ duplo-lúmen ☐ nasotraqueal ☐ bloqueador brônquico ☐ endobrônquico ☐		
Tipo de anestesia	venosa total ☐ inalatória ☐ balanceada ☐		
Antibioticoprofilaxia	sim ☐ não ☐		
Ventilação monopulmonar	sim ☐ não ☐	se sim	pulmão ventilado: D ☐ E ☐ duração (min): ________
Bloqueio do neuroeixo	sim ☐ não ☐	se sim	peridural ☐ raquianestesia ☐ duplo bloqueio ☐
Monitorização neuromuscular	sim ☐ não ☐	se sim	EMG ☐ MMG ☐ AMG ☐
Uso de Trendelenburg	sim ☐ não ☐	se sim	normal ☐ acentuado (≥40°) ☐
Mudança para técnica aberta	sim ☐ não ☐		
Mudança para laparoscopia	sim ☐ não ☐		
Insuflação de CO_2	sim ☐ não ☐	se sim	abdominal ☐ torácica ☐ mediastino ☐
Duração da anestesia (min): ________ da intubação à extubação (ou saída do centro cirúrgico se em ventilação mecânica)			
Duração da cirurgia (min): ________ da incisão ao fechamento			
Drogas, fluídos e transfusão			
Cristaloide	sim ☐ não ☐	se sim	quantidade total (mL): ________
Coloide sintético	sim ☐ não ☐	se sim	quantidade total (mL): ________
Albumina	sim ☐ não ☐	se sim	quantidade total (mL): ________
Concentrado de hemácias	sim ☐ não ☐	se sim	quantidade total (un): ________
Opioides	sim ☐ não ☐	se sim	curta duração ☐ longa duração ☐
Bloqueador neuromuscular	sim ☐ não ☐	se sim	Rocurônio ☐ Vecurônio ☐ Atracúrio ☐ Cisatracúrio ☐ Pancurônio ☐
Reversão farmacológica do bloqueio neuromuscular	sim ☐ não ☐	se sim	Sugammadex ☐ Neostigmine ☐ Piridostigmine ☐ Fisostigmine ☐
Final da anestesia			
Perda sanguínea estimada	sim ☐ não ☐	se sim	quantidade total (mL): ________
Débito urinário	sim ☐ não ☐	se sim	quantidade total (mL): ________
Curarização residual	sim ☐ não ☐		
Temperatura	sim ☐ não ☐	se sim	valor (°C): ________
Peridural pós-operatória	sim ☐ não ☐		
Complicações no intraoperatório			
Dessaturação (SpO_2 <92% por 3 minutos ou mais)			sim ☐ não ☐
Manobra de recrutamento não planejada previamente			sim ☐ não ☐
Redução de pressão na via aérea			sim ☐ não ☐
Hipotensão (PAS <90mmHg ou PAM <65mmHg por 3 minutos ou mais)			sim ☐ não ☐
Arritmia aguda (FA, TV, TSV ou FV)			sim ☐ não ☐
Necessidade de droga vasoativa não prevista e de forma contínua			sim ☐ não ☐

2. DEFINIÇÕES

Pulmão ventilado: D (direito) ou E (esquerdo)	EMG: eletromiografia
MMG: mecanografia	AMG: aceleromiografia
mL: mililitros	UN: unidades
SpO_2: oximetria de pulso	PAs: pressão arterial sistólica
PAm: pressão arterial média	FA: fibrilação atrial
TV: taquicardia ventricular	TSV: taquicardia supraventricular
FV: fibrilação ventricular	°C: graus Celsius
Min: minutos	

Curarização residual
definido como uma taxa de estimulação no *train-of-four* (TOF) <0,9 ou diagnosticada clinicamente

Opioides
Curta duração: alfentanil, fentanil, sufentanil, remifentanil, morfina
Longa duração: opioides em formulação de liberação estendida ou prolongada

Dessaturação
definida como SpO_2 <92% por 3 minutos ou mais

Hipotensão
definida como pressão arterial sistólica <90mmHg ou pressão arterial média <65mmHg por 3 minutos ou mais

continua...

...Continuação

Anexo 1

2. DEFINIÇÕES

Arritmia

Fibrilação atrial (FA): definida por irregularidade absoluta de intervalos R-R e perda simultânea de ondas P identificáveis nas gravações do ECG

Taquicardia ventricular sustentada (TV): caracterizada por ≥3 complexos QRS consecutivos com um complexo QRS largo a uma FC >100 batimentos/min e duração >30 segundos

Taquicardia supraventricular (TSV): identificada como um complexo QRS estreito (<0,12 segundo) e uma FC >180 batimentos/min

Fibrilação ventricular (FV): definida como a descarga eléctrica ventricular caótica, com acentuada variabilidade de morfologia, amplitude e ciclo de QRS

Necessidade de droga vasoativa

Qualquer necessidade de droga vasoativa não prevista e de forma contínua. Drogas consideradas: fenilefrina, vasopressina, dopamina, norepinefrina, epinefrina, dobutamina, efedrina, atropina e/ou milrinone

3. PARÂMETROS VENTILATÓRIOS

	T_1	T_2	T_3	$T_{4.1}$	$T_{4.2}$	$T_{4.3}$	$T_{4.4}$	$T_{4.5}$	$T_{4.6}$	$T_{4.7}$	$T_{4.8}$	$T_{4.9}$	$T_{4.10}$	T_5
Parâmetros ventilatórios														
Modo	PC ☐<br>VC ☐	PC ☐<br>VC ☐	PC ☐<br>VC ☐	PC ☐<br>VC ☐	PC ☐<br>VC ☐	PC ☐<br>VC ☐	PC ☐<br>VC ☐	PC ☐<br>VC ☐	PC ☐<br>VC ☐	PC ☐<br>VC ☐	PC ☐<br>VC ☐	PC ☐<br>VC ☐	PC ☐<br>VC ☐	PC ☐<br>VC ☐
Ppico (cmH_2O)														
Pplato (cmH_2O)														
Pmédia (cmH_2O)														
PEEP (cmH_2O)														
V_T inspirado (mL)														
FR (/min)														
I:E														
FiO_2 (%)														
MR	PEEP ☐<br>V_T ☐<br>Dupla ☐<br>Bag ☐<br>CPAP ☐<br>Não ☐	PEEP ☐<br>V_T ☐<br>Dupla ☐<br>Bag ☐<br>CPAP ☐<br>Não ☐	PEEP ☐<br>V_T ☐<br>Dupla ☐<br>Bag ☐<br>CPAP ☐<br>Não ☐	PEEP ☐<br>V_T ☐<br>Dupla ☐<br>Bag ☐<br>CPAP ☐<br>Não ☐	PEEP ☐<br>V_T ☐<br>Dupla ☐<br>Bag ☐<br>CPAP ☐<br>Não ☐	PEEP ☐<br>V_T ☐<br>Dupla ☐<br>Bag ☐<br>CPAP ☐<br>Não ☐	PEEP ☐<br>V_T ☐<br>Dupla ☐<br>Bag ☐<br>CPAP ☐<br>Não ☐	PEEP ☐<br>V_T ☐<br>Dupla ☐<br>Bag ☐<br>CPAP ☐<br>Não ☐	PEEP ☐<br>V_T ☐<br>Dupla ☐<br>Bag ☐<br>CPAP ☐<br>Não ☐	PEEP ☐<br>V_T ☐<br>Dupla ☐<br>Bag ☐<br>CPAP ☐<br>Não ☐	PEEP ☐<br>V_T ☐<br>Dupla ☐<br>Bag ☐<br>CPAP ☐<br>Não ☐	PEEP ☐<br>V_T ☐<br>Dupla ☐<br>Bag ☐<br>CPAP ☐<br>Não ☐	PEEP ☐<br>V_T ☐<br>Dupla ☐<br>Bag ☐<br>CPAP ☐<br>Não ☐	PEEP ☐<br>V_T ☐<br>Dupla ☐<br>Bag ☐<br>CPAP ☐<br>Não ☐
Parâmetros vitais														
SpO_2 (%)														
$etCO_2$ (mmHg)														
PAm (mmHg)														
FC (/min)														
Outros														
Ppneumo (mmHg)<br>Maior valor na hora														
Posicionamento	DDH ☐<br>DVH ☐<br>DL ☐<br>LIT ☐<br>CDV ☐<br>CAV ☐<br>CP ☐	DDH ☐<br>DVH ☐<br>DL ☐<br>LIT ☐<br>CDV ☐<br>CAV ☐<br>CP ☐	DDH ☐<br>DVH ☐<br>DL ☐<br>LIT ☐<br>CDV ☐<br>CAV ☐<br>CP ☐	DDH ☐<br>DVH ☐<br>DL ☐<br>LIT ☐<br>CDV ☐<br>CAV ☐<br>CP ☐	DDH ☐<br>DVH ☐<br>DL ☐<br>LIT ☐<br>CDV ☐<br>CAV ☐<br>CP ☐	DDH ☐<br>DVH ☐<br>DL ☐<br>LIT ☐<br>CDV ☐<br>CAV ☐<br>CP ☐	DDH ☐<br>DVH ☐<br>DL ☐<br>LIT ☐<br>CDV ☐<br>CAV ☐<br>CP ☐	DDH ☐<br>DVH ☐<br>DL ☐<br>LIT ☐<br>CDV ☐<br>CAV ☐<br>CP ☐	DDH ☐<br>DVH ☐<br>DL ☐<br>LIT ☐<br>CDV ☐<br>CAV ☐<br>CP ☐	DDH ☐<br>DVH ☐<br>DL ☐<br>LIT ☐<br>CDV ☐<br>CAV ☐<br>CP ☐	DDH ☐<br>DVH ☐<br>DL ☐<br>LIT ☐<br>CDV ☐<br>CAV ☐<br>CP ☐	DDH ☐<br>DVH ☐<br>DL ☐<br>LIT ☐<br>CDV ☐<br>CAV ☐<br>CP ☐	DDH ☐<br>DVH ☐<br>DL ☐<br>LIT ☐<br>CDV ☐<br>CAV ☐<br>CP ☐	DDH ☐<br>DVH ☐<br>DL ☐<br>LIT ☐<br>CDV ☐<br>CAV ☐<br>CP ☐

4. DEFINIÇÕES

Ppico: pressão de pico

Pmédia: pressão média

V_T: volume corrente

I:E: relação inspiração:expiração

$etCO_2$: CO_2 exalado ao final da expiração

FC: frequência cardíaca

Pplato: pressão de platô

A pressão de platô deverá ser medida com o uso de uma pausa inspiratória de pelo menos 0,5 segundo

PEEP: pressão positiva ao final da expiração

FR: frequência respiratória

FiO_2: fração inspirada de oxigênio

SpO_2: oximetria de pulso

PAM: pressão arterial média

Ppneumo: pressão do pneumoperitôneo

MR: manobra de recrutamento alveolar

PEEP: aumento gradual da PEEP com volume corrente constante

V_T: aumento gradual do volume com PEEP constante

Dupla: PEEP e volume corrente são ambos gradual aumentados

Bag: hiperinflação manual com balão/ saco

CPAP: pressão positiva nas vias aéreas superior a $30cmH_2O$ aplicada durante 10 a 30 segundos

continua...

...Continuação

Anexo 1

4. DEFINIÇÕES

Modo: modo ventilatório
- PC: pressão controlada
- VC: volume controlado

Posicionamento: posicionamento durante o ato cirúrgico
- DDH: decúbito dorsal horizontal (supino)
- DVH: decúbito ventral horizontal (prona)
- DL: decúbito lateral
- LIT: litotomia
- CDV: cefalodeclive (Trendelenburg)
- CAV: cefaloaclive (Trendelenburg reverso)
- CP: sentado

T: momentos cirúrgicos
- 1: 5 minutos após início da ventilação mecânica
- 2: 5 minutos após realização do pneumoperitôneo (não preencher se não realizado)
- 3: 5 minutos após posicionamento intraoperatório definitivo
- 4.1: 60 minutos após T_3
- 4.2-4.10: a cada 60 minutos
- 5: 5 minutos após desinsuflação do pneumoperitôneo (se realizado) e posicionamento final

1. VISITA PÓS-OPERATÓRIA NO DIA 0 (FINAL DA CIRURGIA ATÉ 23h59)

Recuperação			
Perda de *follow-up*	sim ☐ não ☐	se sim	alta ☐ óbito ☐ transferência ☐ outro ☐: ________
Continuação de VM após cirurgia	sim ☐ não ☐	se sim	planejada ☐ não planejada (contínua) ☐ não planejada (reintubação) ☐
Se reintubação, causa	IRpA ☐ RNC ☐ Instabilidade hemodinâmica ☐		
Admissão a UTI após cirurgia	sim ☐ não ☐	se sim	planejada ☐ não planejada ☐
Complicações pulmonares no pós-operatório			
Necessidade de oxigênio<br>PaO_2 <60mmHg ou SpO_2 <90% em AA	sim ☐ não ☐	se sim	FiO_2 (%) ofertada: ________
Insuficiência respiratória aguda<br>PaO_2 <60mmHg ou SpO_2 <90% com oxigênio ou necessidade de VNI	sim ☐ não ☐	se sim	Uso de VNI: sim ☐ não ☐ se sim<br>Interface: mascara ☐ helmet ☐
Pneumonia<br>novo/piora infiltrado + 2: febre, leucocitose/leucopenia, secreção purulenta, antibiótico	sim ☐ não ☐		
SDRA<br>de acordo com os critérios de Berlin	sim ☐ não ☐	se sim	leve ☐ moderada ☐ grave ☐
Pneumotórax<br>ar entre a pleura visceral e parietal	sim ☐ não ☐		

2. DEFINIÇÕES

VM: ventilação mecânica

FiO_2: fração inspirada de oxigênio

PaO_2: pressão parcial de oxigênio

AA: ar ambiente

IRpA: insuficiência respiratória aguda

UTI: unidade de terapia intensiva

VNI: ventilação não invasiva

SpO_2: oximetria de pulso

SDRA: síndrome do desconforto respiratório agudo

RNC: rebaixamento do nível de consciência

Necessidade de oxigênio
- definido como oxigênio suplementar utilizado devido *à* PaO_2 <60mmHg ou SpO_2 <92% em ar ambiente (em indivíduos sem doença pulmonar prévia) ou SpO_2 <88% (em indivíduos com doença pulmonar prévia)

Insuficiência respiratória aguda
- definida como PaO_2 <60mmHg ou SpO_2 <92%, apesar da terapia com oxigênio, ou necessidade de ventilação mecânica não invasiva (VNI)

Pneumonia
- definida pela presença de um infiltrado radiográfico novo ou progressivo, além de pelo menos duas das quatro características clínicas: febre >38°*C,* leucocitose ou leucopenia (contagem de leucócitos >12.000 células/mm ou <*4*.000 células/mm^3), secreção purulenta ou uso de antibiótico

Síndrome do Desconforto Respiratório Agudo (SDRA)
- Tempo: dentro de uma semana de um insulto clínico conhecido ou piora dos sintomas respiratórios
- Imagem: opacidades bilaterais não explicadas totalmente por derrames pleurais, colapso pulmonar ou lobar ou nódulos (radiografia de tórax ou tomografia computadorizada)
- Origem do edema: Insuficiência respiratória não explicada totalmente por falência cardíaca ou sobrecarga volêmica. Necessita de avaliação objetiva (exemplo: ecocardiograma) para excluir edema hidrostático se não houver nenhum fator de risco presente
- Oxigenação:
  - Leve: 200mmHg $<PaO_2/FiO_2 \leq$300mmHg com PEEP ou CPAP ≥5cmH2O (pode ser via VNI)
  - Moderada: 100mmHg$<PaO_2/FiO_2 \leq$200mmHg com PEEP
  - Grave: 100mmHg$\leq PaO_2/FiO_2$ com PEEP

Pneumotórax
- definido como a presença de ar entre a pleura visceral e parietal, o diagnóstico pode ser feito por exame clínico e RX tórax

continua...

...Continuação

Anexo 1

1. VISITA PÓS-OPERATÓRIA NO DIA 1 (DA 0h00 ATÉ 23h59)			
Recuperação			
Perda de *follow-up*	sim ☐ não ☐	se sim	alta ☐ óbito ☐ transferência ☐ outro ☐: ________
Necessidade de VM nova	sim ☐ não ☐		
Necessidade de admissão em UTI	sim ☐ não ☐		
Complicações pulmonares no pós-operatório			
Necessidade de oxigênio<br>PaO_2 <60mmHg ou SpO_2 <90% em AA	sim ☐ não ☐	se sim	FiO_2 (%) ofertada: ________
Insuficiência respiratória aguda<br>PaO_2 <60mmHg ou SpO_2 <90% com oxigênio ou necessidade de VNI	sim ☐ não ☐	se sim	Uso de VNI: sim ☐ não ☐ se sim<br>Interface: máscara ☐ *helmet* ☐
Pneumonia<br>novo/piora infiltrado + 2: febre, leucocitose/leucopenia, secreção purulenta, antibiótico	sim ☐ não ☐		
SDRA<br>de acordo com os critérios de Berlin	sim ☐ não ☐	se sim	leve ☐ moderada ☐ grave ☐
Pneumotórax<br>ar entre a pleura visceral e parietal	sim ☐ não ☐		

2. DEFINIÇÕES

VM: ventilação mecânica	UTI: unidade de terapia intensiva
FiO_2: fração inspirada de oxigênio	VNI: ventilação não invasiva
PaO_2: pressão parcial de oxigênio	SpO_2: oximetria de pulso
AA: ar ambiente	SDRA: síndrome do desconforto respiratório agudo

Necessidade de oxigênio

definido como oxigênio suplementar utilizado devido à PaO_2 <60mmHg ou SpO_2 <92% em ar ambiente (em indivíduos sem doença pulmonar prévia) ou SpO_2 <88% (em indivíduos com doença pulmonar prévia)

Insuficiência respiratória aguda

definida como PaO_2 <60mmHg ou SpO_2 <92%, apesar da terapia com oxigênio, ou necessidade de ventilação mecânica não invasiva (VNI)

Pneumonia

definida pela presença de um infiltrado radiográfico novo ou progressivo, além de pelo menos duas das quatro características clínicas: febre >38°C, leucocitose ou leucopenia (contagem de leucócitos >12.000 células/mm ou <4.000 células/mm^3), secreção purulenta ou uso de antibiótico

Síndrome do Desconforto Respiratório Agudo (SDRA)

Tempo: dentro de uma semana de um insulto clínico conhecido ou piora dos sintomas respiratórios

Imagem: opacidades bilaterais não explicadas totalmente por derrames pleurais, colapso pulmonar ou lobar ou nódulos (radiografia de tórax ou tomografia computadorizada)

Origem do edema: insuficiência respiratória não explicada totalmente por falência cardíaca ou sobrecarga volêmica. Necessita de avaliação objetiva (exemplo: ecocardiograma) para excluir edema hidrostático se não houver nenhum fator de risco presente

Oxigenação:

Leve: 200mmHg $<PaO_2/FiO_2 \leq$ 300mmHg com PEEP ou CPAP $\geq$5cmH_2O (pode ser via VNI)

Moderada: 100mmHg $<PaO_2/FiO_2 \leq$ 200mmHg com PEEP

Grave: 100mmHg $\leq PaO_2/FiO_2$ com PEEP

Pneumotórax

definido como a presença de ar entre a pleura visceral e parietal, o diagnóstico pode ser feito por exame clínico e raio X de tórax

1. VISITA PÓS-OPERATÓRIA NO DIA 2 (DA 0h00 ATÉ 23h59)			
Recuperação			
Perda de *follow-up*	sim ☐ não ☐	se sim	alta ☐ óbito ☐ transferência ☐ outro ☐: ________
Necessidade de VM nova	sim ☐ não ☐		
Necessidade de admissão em UTI	sim ☐ não ☐		
Complicações pulmonares no pós-operatório			
Necessidade de oxigênio<br>PaO_2 <60mmHg ou SpO_2 <90% em AA	sim ☐ não ☐	se sim	FiO_2 (%) ofertada: ________
Insuficiência respiratória aguda<br>PaO_2 <60mmHg ou SpO_2 <90% com oxigênio ou necessidade de VNI	sim ☐ não ☐	se sim	Uso de VNI: sim ☐ não ☐ se sim<br>Interface: máscara ☐ *helmet* ☐
Pneumonia<br>novo/piora infiltrado + 2: febre, leucocitose/leucopenia, secreção purulenta, antibiótico	sim ☐ não ☐		
SDRA<br>de acordo com os critérios de Berlin	sim ☐ não ☐	se sim	leve ☐ moderada ☐ grave ☐
Pneumotórax<br>ar entre a pleura visceral e parietal	sim ☐ não ☐		

continua...

...Continuação

Anexo 1

2. DEFINIÇÕES

VM: ventilação mecânica	UTI: unidade de terapia intensiva
FiO_2: fração inspirada de oxigênio	VNI: ventilação não invasiva
PaO_2: pressão parcial de oxigênio	SpO_2: oximetria de pulso
AA: ar ambiente	SDRA: síndrome do desconforto respiratório agudo

Necessidade de oxigênio
definido como oxigênio suplementar utilizado devido à PaO_2 <60mmHg ou SpO_2 <92% em ar ambiente (em indivíduos sem doença pulmonar prévia) ou SpO_2 <88% (em indivíduos com doença pulmonar prévia)

Insuficiência respiratória aguda
definida como PaO_2 <60mmHg ou SpO_2 <92%, apesar da terapia com oxigênio, ou necessidade de ventilação mecânica não invasiva (VNI)

Pneumonia
definida pela presença de um infiltrado radiográfico nsovo ou progressivo, além de pelo menos duas das quatro características clínicas: febre >38°C, leucocitose ou leucopenia (contagem de leucócitos >12.000 células/mm ou <4.000 células/mm^3), secreção purulenta ou uso de antibiótico

Síndrome do Desconforto Respiratório Agudo (SDRA)
Tempo: dentro de uma semana de um insulto clínico conhecido ou piora dos sintomas respiratórios
Imagem: opacidades bilaterais não explicadas totalmente por derrames pleurais, colapso pulmonar ou lobar ou nódulos (radiografia de tórax ou tomografia computadorizada)
Origem do edema: insuficiência respiratória não explicada totalmente por falência cardíaca ou sobrecarga volêmica. Necessita de avaliação objetiva (exemplo: ecocardiograma) para excluir edema hidrostático se não houver nenhum fator de risco presente
Oxigenação:
Leve: 200mmHg $<PaO_2/FiO_2 \leq$300mmHg com PEEP ou CPAP $\geq$5cmH_2O (pode ser via VNI)
Moderada: 100mmHg $<PaO_2/FiO_2 \leq$200mmHg com PEEP
Grave: 100mmHg $\leq PaO_2/FiO_2$ com PEEP

Pneumotórax
definido como a presença de ar entre a pleura visceral e parietal, o diagnóstico pode ser feito por exame clínico e raio X de tórax

1. VISITA PÓS-OPERATÓRIA NO DIA 3 (DA 0h00 ATÉ 23h59)

Recuperação

Perda de *follow-up*	sim ☐ não ☐	se sim	alta ☐ óbito ☐ transferência ☐ outro ☐: ________
Necessidade de VM nova	sim ☐ não ☐		
Necessidade de admissão em UTI	sim ☐ não ☐		

Complicações Pulmonares no Pós-Operatório

Necessidade de oxigênio<br>PaO_2 <60mmHg ou SpO_2 <90% em AA	sim ☐ não ☐	se sim	FiO_2 (%) ofertada: ________
Insuficiência respiratória aguda<br>PaO_2 <60mmHg ou SpO_2 <90% com oxigênio ou necessidade de VNI	sim ☐ não ☐	se sim	Uso de VNI: sim ☐ não ☐ se sim<br>Interface: máscara ☐ *helmet* ☐
Pneumonia<br>novo/piora infiltrado + 2: febre, leucocitose/leucopenia, secreção purulenta, antibiótico	sim ☐ não ☐		
SDRA<br>de acordo com os critérios de Berlin	sim ☐ não ☐	se sim	leve ☐ moderada ☐ grave ☐
Pneumotórax<br>ar entre a pleura visceral e parietal	sim ☐ não ☐		

2. DEFINIÇÕES

VM: ventilação mecânica	UTI: unidade de terapia intensiva
FiO_2: fração inspirada de oxigênio	VNI: ventilação não invasiva
PaO_2: pressão parcial de oxigênio	SpO_2: oximetria de pulso
AA: ar ambiente	SDRA: síndrome do desconforto respiratório agudo

Necessidade de oxigênio
definido como oxigênio suplementar utilizado devido à PaO_2 <60mmHg ou SpO_2 <92% em ar ambiente (em indivíduos sem doença pulmonar prévia) ou SpO_2 <88% (em indivíduos com doença pulmonar prévia)

Insuficiência respiratória aguda
definida como PaO_2 <60mmHg ou SpO_2 <92%, apesar da terapia com oxigênio, ou necessidade de ventilação mecânica não invasiva (VNI)

Pneumonia
definida pela presença de um infiltrado radiográfico novo ou progressivo, além de pelo menos duas das quatro características clínicas: febre >38°C, leucocitose ou leucopenia (contagem de leucócitos >12.000 células/mm ou <4.000 células/mm^3), secreção purulenta ou uso de antibiótico

continua...

...Continuação

Anexo 1

2. DEFINIÇÕES

Síndrome do Desconforto Respiratório Agudo (SDRA)

Tempo: dentro de uma semana de um insulto clínico conhecido ou piora dos sintomas respiratórios

Imagem: opacidades bilaterais não explicadas totalmente por derrames pleurais, colapso pulmonar ou lobar ou nódulos (radiografia de tórax ou tomografia computadorizada)

Origem do edema: insuficiência respiratória não explicada totalmente por falência cardíaca ou sobrecarga volêmica. Necessita de avaliação objetiva (exemplo: ecocardiograma) para excluir edema hidrostático se não houver nenhum fator de risco presente

Oxigenação:

Leve: 200mmHg $<PaO_2/FiO_2 \leq$300mmHg com PEEP ou CPAP $\geq$5cmH_2O (pode ser via VNI)

Moderada: 100mmHg $<PaO_2/FiO_2 \leq$200mmHg com PEEP

Grave: 100mmHg $\leq PaO_2/FiO_2$ com PEEP

Pneumotórax

definido como a presença de ar entre a pleura visceral e parietal, o diagnóstico pode ser feito por exame clínico e raio X de tórax

1. VISITA PÓS-OPERATÓRIA NO DIA 4 (DA 0h00 ATÉ 23h59)

Recuperação

Perda de *follow-up*	sim ☐ não ☐	se sim	alta ☐ óbito ☐ transferência ☐ outro ☐: ________
Necessidade de VM nova	sim ☐ não ☐		
Necessidade de admissão em UTI	sim ☐ não ☐		

Complicações pulmonares no pós-operatório

Necessidade de oxigênio<br>PaO_2 <60mmHg ou SpO_2 <90% em AA	sim ☐ não ☐	se sim	FiO_2 (%) ofertada: ________
Insuficiência respiratória aguda<br>PaO_2 <60mmHg ou SpO_2 <90% com oxigênio ou necessidade de VNI	sim ☐ não ☐	se sim	Uso de VNI: sim ☐ não ☐ se sim<br>Interface: máscara ☐ *helmet* ☐
Pneumonia<br>novo/piora infiltrado + 2: febre, leucocitose/leucopenia, secreção purulenta, antibiótico	sim ☐ não ☐		
SDRA<br>de acordo com os critérios de Berlin	sim ☐ não ☐	se sim	leve ☐ moderada ☐ grave ☐
Pneumotórax<br>ar entre a pleura visceral e parietal	sim ☐ não ☐		

2. DEFINIÇÕES

VM: ventilação mecânica

FiO_2: fração inspirada de oxigênio

PaO_2: pressão parcial de oxigênio

AA: ar ambiente

UTI: unidade de terapia intensiva

VNI: ventilação não invasiva

SpO_2: oximetria de pulso

SDRA: síndrome do desconforto respiratório agudo

Necessidade de oxigênio

definido como oxigênio suplementar utilizado devido à PaO_2 <60mmHg ou SpO_2 <92% em ar ambiente (em indivíduos sem doença pulmonar prévia) ou SpO_2 <88% (em indivíduos com doença pulmonar prévia)

Insuficiência respiratória aguda

definida como PaO_2 <60mmHg ou SpO_2 <92%, apesar da terapia com oxigênio, ou necessidade de ventilação mecânica não invasiva (VNI)

Pneumonia

definida pela presença de um infiltrado radiográfico novo ou progressivo, além de pelo menos duas das quatro características clínicas: febre >38°C, leucocitose ou leucopenia (contagem de leucócitos >12.000 células/mm ou <4.000 células/mm^3), secreção purulenta ou uso de antibiótico

Síndrome do Desconforto Respiratório Agudo (SDRA)

Tempo: dentro de uma semana de um insulto clínico conhecido ou piora dos sintomas respiratórios

Imagem: opacidades bilaterais não explicadas totalmente por derrames pleurais, colapso pulmonar ou lobar ou nódulos (radiografia de tórax ou tomografia computadorizada)

Origem do edema: insuficiência respiratória não explicada totalmente por falência cardíaca ou sobrecarga volêmica. Necessita de avaliação objetiva (exemplo: ecocardiograma) para excluir edema hidrostático se não houver nenhum fator de risco presente

Oxigenação:

Leve: 200mmHg $<PaO_2/FiO_2 \leq$300mmHg com PEEP ou CPAP $\geq$5cmH_2O (pode ser via VNI)

Moderada: 100mmHg $<PaO_2/FiO_2 \leq$200mmHg com PEEP

Grave: 100mmHg $\leq PaO_2/FiO_2$ com PEEP

Pneumotórax

definido como a presença de ar entre a pleura visceral e parietal, o diagnóstico pode ser feito por exame clínico e raio X de tórax

continua...

...Continuação

Anexo 1

1. VISITA PÓS-OPERATÓRIA NO DIA 5 (DA 0h00 ATÉ 23h59)

Recuperação

Perda de *follow-up* sim ☐ não ☐ se sim alta ☐ óbito ☐ transferência ☐ outro ☐: _________

Necessidade de VM nova sim ☐ não ☐

Necessidade de admissão a UTI sim ☐ não ☐

Complicações pulmonares no pós-operatório

Necessidade de oxigênio sim ☐ não ☐ se sim FiO_2 (%) ofertada: _________
PaO_2 <60mmHg ou SpO_2 <90% em AA

Insuficiência respiratória aguda sim ☐ não ☐ se sim Uso de VNI: sim ☐ não ☐ se sim
PaO_2 <60mmHg ou SpO_2 <90% com oxigênio ou necessidade de VNI Interface: máscara ☐ *helmet* ☐

Pneumonia sim ☐ não ☐
novo/piora infiltrado + 2: febre, leucocitose/leucopenia, secreção purulenta, antibiótico

SDRA sim ☐ não ☐ se sim leve ☐ moderada ☐ grave ☐
de acordo com os critérios de Berlin

Pneumotórax sim ☐ não ☐
ar entre a pleura visceral e parietal

2. DEFINIÇÕES

VM: ventilação mecânica
UTI: unidade de terapia intensiva
FiO_2: fração inspirada de oxigênio
VNI: ventilação não invasiva
PaO_2: pressão parcial de oxigênio
SpO_2: oximetria de pulso
AA: ar ambiente
SDRA: síndrome do desconforto respiratório agudo

Necessidade de oxigênio
definido como oxigênio suplementar utilizado devido à PaO_2 <60mmHg ou SpO_2 <92% em ar ambiente (em indivíduos sem doença pulmonar prévia) ou SpO_2 <88% (em indivíduos com doença pulmonar prévia)

Insuficiência respiratória aguda
definida como PaO_2 <60mmHg ou SpO_2 <92%, apesar da terapia com oxigênio, ou necessidade de ventilação mecânica não invasiva (VNI)

Pneumonia
definida pela presença de um infiltrado radiográfico novo ou progressivo, além de pelo menos duas das quatro características clínicas: febre >38°C, leucocitose ou leucopenia (contagem de leucócitos >12.000 células/mm ou <4.000 células/mm^3), secreção purulenta ou uso de antibiótico

Síndrome do Desconforto Respiratório Agudo (SDRA)
Tempo: dentro de uma semana de um insulto clínico conhecido ou piora dos sintomas respiratórios
Imagem: opacidades bilaterais não explicadas totalmente por derrames pleurais, colapso pulmonar ou lobar ou nódulos (radiografia de tórax ou tomografia computadorizada)
Origem do edema: insuficiência respiratória não explicada totalmente por falência cardíaca ou sobrecarga volêmica. Necessita de avaliação objetiva (exemplo: ecocardiograma) para excluir edema hidrostático se não houver nenhum fator de risco presente
Oxigenação:
Leve: 200mmHg $<PaO_2/FiO_2 \leq$300mmHg com PEEP ou CPAP ≥5cmH_2O (pode ser via VNI)
Moderada: 100mmHg $<PaO_2/FiO_2 \leq$200mmHg com PEEP
Grave: 100mmHg $\leq PaO_2/FiO_2$ com PEEP

Pneumotórax
definido como a presença de ar entre a pleura visceral e parietal, o diagnóstico pode ser feito por exame clínico e raio X de tórax

1. VISITA DE ALTA (DIA DA ALTA)

Desfechos

Data da admissão: ___ / ___ / _____ Data da alta: ___ / ___ / _____

Tempo de internação: _______ dias

Óbito durante internação sim ☐ não ☐ se sim Data do óbito: ___ / ___ / _____

Comentários:
