# Supplementary material for: Methodological description of clinical research data collection through electronic medical records in a center participating in an international multicenter study
Source: Einstein (Sao Paulo). 2019 Sep 16;17(4):eAE4791. doi: 10.31744/einstein_journal/2019AE4791 (PMC6748344; doi:10.31744/einstein_journal/2019AE4791)
Supplement: Translation

# Translation: Abordagem e descrição metodológica do uso do prontuário eletrônico como forma de coleta de dados em pesquisa clínica em centro participante de estudo internacional e multicêntrico

**Authors:** Veronica Neves Fialho Queiroz, Andrea da Costa Moreira de Oliveira, Renato Carneiro de Freitas Chaves, Lucas Araújo de Borges Moura, Daniel Sousa César, Flávio Takaoka, Ary Serpa

**Affiliations:** 1 Hospital Israelita Albert Einstein, São Paulo, SP, Brasil.; 2 Irmandade da Santa Casa de Misericórdia de Santos, Santos, SP, Brasil.; 3 Department of Intensive Care Medicine, University Medical Centers, Amsterdam University, Amsterdam, Netherlands.

**Keywords:** Registros eletrônicos de saúde, Confiabilidade dos dados, Coleta de dados, Anestesia, Cuidados críticos

## Abstract

A coleta de dados para pesquisa clínica pode representar um desafio em que sistemas de registro eletrônico em saúde podem facilitar o processo. O objetivo deste estudo foi descrever e avaliar o uso secundário de registros eletrônicos em saúde na coleta de dados para um estudo clínico observacional. Usamos o Cerner Millennium^®^, software de registro eletrônico em saúde, de acordo com os seguintes passos: (1) cruzamento dos dados das fichas de coleta de dados do estudo e dos registros eletrônicos em saúde; (2) desenvolvimento de método para coleta manual de dados não registrados no Cerner Millennium^®^; (3) desenvolvimento de interface de estudo para a coleta automática de dados nos registros eletrônicos em saúde; (4) treinamento de colaboradores; (5) avaliação da qualidade dos dados; e (6) preenchimento da ficha eletrônica de coleta de dados no fim do estudo. Três fichas de coleta de dados foram consolidadas em uma ficha eletrônica de coleta de dados no fim do estudo. Os pesquisadores realizaram análise qualitativa e quantitativa de dados diariamente. Foram coletados dados de 94 pacientes. Na primeira ficha de coleta de dados, 76,5% das variáveis foram obtidas eletronicamente, na segunda, 95,5%, e na terceira, 100%. A avaliação diária de qualidade do processo como um todo revelou dados completos e corretos, ampla adesão dos colaboradores e mínima interferência na prática profissional. O uso secundário dos registros eletrônicos em saúde é seguro e efetivo, reduz o trabalho manual e produz dados confiáveis. O cuidado anestésico ao paciente e a coleta de dados podem ser realizados simultaneamente pelo mesmo professional.

## INTRODUÇÃO

A contínua informatização dos registros médicos por meio da implementação de softwares de Registros Eletrônicos em Saúde (RES) torna possível a constante compilação de dados obtidos durante o cuidado, produzindo grandes bancos de dados secundários. A intersecção entre as informações requeridas em estudos clínicos com aquelas contidas nas informações comumente coletadas no cuidado diário do paciente e registradas nos RES^1^ estimulou o surgimento de novas formas de coletas de dados para a pesquisa clínica, utilizando o RES como importante ferramenta neste processo.^2^

A anestesia demanda atenção constante ao paciente, o que torna os registros anestésicos manuais de difícil realização, resultando frequentemente em fichas com dados incompletos e, muitas vezes, imprecisos.^3^ Os dispositivos que possibilitam a migração automática de dados de monitorização intraoperatória para a ficha de anestesia eletrônica são recursos disponíveis nos RES, possibilitando coleta de dados mais acurada, prevenindo a perda de informações clínicas e reduzindo o trabalho manual do profissional nesta etapa.^4^ Sem estes recursos, a colaboração em atividades de pesquisa concomitante ao manejo anestésico é difícil, e dispor de um profissional exclusivo para a coleta de dados é oneroso e impraticável em grande parte dos hospitais. Assim, o uso dos RES no auxílio a pesquisa clínica torna-se opção atrativa, principalmente no cenário intraoperatório.

No presente estudo, temos como objetivo primário descrever uma metodologia desenvolvida para integrar um sistema de RES à ficha de coleta de dados (CRF) de um estudo clínico realizado durante o período intraoperatório, possibilitando a coleta de dados de forma automática. Ainda, os objetivos secundários foram quantificar os dados obtidos por esta coleta automática e avaliar a qualidade dos mesmos.

## MÉTODOS

Este estudo foi realizado em novembro de 2017, no Hospital Israelita Albert Einstein (HIAE), em São Paulo (SP), Brasil.

### Desenho do estudo *Assessment of Ventilatory Management During General Anesthesia for Robotic Surgery and Its Effects on Postoperative Pulmonary Complications*

O AVATaR^5^ (*Assessment of Ventilatory Management During General Anesthesia for Robotic Surgery and Its Effects on Postoperative Pulmonary Complications*) é um estudo prospectivo, observacional e multicêntrico. O objetivo primário é avaliar a incidência de complicações pulmonares no pós-operatório (CPP) em pacientes submetidos à ventilação mecânica (VM) para cirurgia robótica. O objetivo secundário é caracterizar as práticas vigentes de VM e sua associação com posicionamento cirúrgico e incidência de CPP (Figura 1). Atualmente o estudo ocorre em 29 centros participantes, mas a metodologia de coleta descrita neste manuscrito diz respeito somente à coleta realizada no HIAE.

**Figura 1:** Fluxo do estudo AVATaR (*Assessment of Ventilatory Management During General Anesthesia for Robotic Surgery and Its Effects on Postoperative Pulmonary Complications)*

CRF: ficha de coleta de dados.

Ainda, o HIAE é o centro coordenador do estudo, e todos os outros centros somente estão envolvidos na inclusão de pacientes e coleta de dado local. Os centros envolvidos no estudo estão descritos na tabela 1.

**Tabela 1:** Centros participantes do estudo AVATaR (*Assessment of Ventilatory Management During General Anesthesia for Robotic Surgery and Its Effects on Postoperative Pulmonary Complications*)

Centro	Cidade	País
*Bradford Teaching Hospitals NHS Foundation Trust*	Bradford	Reino Unido
*Cambridge University Hospitals NHS Foundation Trust*	Cambridge	Reino Unido
*Citta della Salute e della Scienza*	Turim	Itália
*City Hospitals Sunderland*	Sunderland	Reino Unido
*Consorcio Hospital General Universitario de Valencia*	Valência	Espanha
*Duesseldorf University Hospital*	Dusseldorf	Alemanha
*East Kent Hospitals University Foundation Trust*	Kent	Reino Unido
*East Lancashire Hospitals NHS Trust*	Lancashire	Reino Unido
*Fondazione Policlinico Universitario Gemelli of Rome*	Roma	Itália
*Hospital Clinic Barcelona*	Barcelona	Espanha
*Hospital Clínico San Carlos*	Madrid	Espanha
Hospital Israelita Albert Einstein	São Paulo	Brasil
Hospital Nove de Julho	São Paulo	Brasil
*Hull and East Yorkshire Hospitals NHS Trust site*	Hull	Reino Unido
*Kliniken Essen-Mitte*	Essen	Alemanha
*Maasstad Ziekenhuis*	Rotterdam	Holanda
*Massachusetts General Hospital*	Boston	Estados Unidos
*Mayo Clinic*	Rochester	Estados Unidos
*MD Anderson Cancer Center*	Houston	Estados Unidos
*Rabin Medical Center Beilinson Hospital*	Petah Tikva	Israel
*Royal Berkshire Hospital*	Reading	Reino Unido
*Royal Hallamshire*	Sheffield	Reino Unido
*Royal Surrey County Hospital NHS Foundation Trust*	Guildford	Reino Unido
*St George’s University Hospitals NHS Foundation Trust*	Tooting	Reino Unido
*Tel Aviv Medical Center*	Tel Aviv	Israel
*Università di Foggia*	Foggia	Itália
*University of California, San Francisco*	San Francisco	Estados Unidos
*University of Genova*	Gênova	Itália
*Wirral University Teaching Hospital NHS Foundation Trust*	Wirral	Reino Unido

O presente estudo foi aprovado pelo Comitê de Ética em Pesquisa do HIAE (CAAE: 67113817.2.1001.007). O Termo de Consentimento Livre e Esclarecido (TCLE) foi obtido de todos os participantes incluídos no estudo. Por fim, a ferramenta descrita foi utilizada somente nos pacientes elegíveis ao estudo e que assinaram o TCLE. Todos os dados coletados são de-identificados quando inseridos no banco de dados final, sendo o acesso a este protegido por senha e restrito ao pesquisador principal e ao estatístico do estudo.

### Ficha de coleta de dados do estudo AVATaR

O CRF final do estudo (Anexo 1) foi dividido em três formulários distintos, de acordo com as fases do período perioperatório: (1) CRF-1, por meio do qual foram coletados dados do pré-operatório; (2) CRF-2, que tratou da coleta dos dados do intraoperatório; e (3) CRF-3, que se voltou à coleta dos dados do pós-operatório.

O CRF-1 é constituído por 77 itens para preenchimento (14 variáveis principais e 63 subtipos das variáveis principais) que referem-se a dados demográficos, sinais vitais basais, status físico e funcional, comorbidades, exames laboratoriais pré-operatórios, complicações respiratórias prévias, tipo de cirurgia, duração esperada do procedimento e topografia da incisão cirúrgica.

O CRF-2 possui duas etapas de coleta. A primeira etapa contém 52 itens para preenchimento (21 variáveis principais e 31 subtipos), que referem-se aos detalhes da anestesia, posicionamento da mesa cirúrgica, local da insuflação do dióxido de carbono, balanço hídrico intraoperatório e duração da cirurgia e da anestesia. A segunda etapa é constituída por detalhes do manejo ventilatório durante o procedimento cirúrgico, obtidos em diferentes momentos: T_1_, 5 minutos após indução anestésica e início da VM ; T_2_, 5 minutos após a insuflação do dióxido de carbono na cavidade abdominal ou torácica; T_3_, 5 minutos após o posicionamento cirúrgico final; T_4_, a cada 60 minutos de tempo decorrido no intraoperatório (subdividido em T_4.1_, T_4.2_, T_4.3_ e assim sucessivamente até o número máximo de 10 horas); e T_5_, 5 minutos após desinsuflação da cavidade e posicionamento final ao término da cirurgia. A segunda etapa do CRF-2 contempla 33 variáveis que são preenchidas no mínimo em 5 momentos, mas, de acordo com o tempo cirúrgico, podem ser preenchidas por até 15 momentos, totalizando 165 variáveis no mínimo ou 495 no máximo.

Por fim, o CRF-3 contém 21 variáveis referentes à recuperação do paciente e ao desenvolvimento de CPP, coletados do dia zero até o dia 5 ou até a alta hospitalar, dependendo do que ocorrer antes.

### Sistema de Registro Eletrônico em Saúde

O HIAE implantou seu RES em janeiro de 2017, e o software utilizado foi o *Cerner Millennium*^®^. O fluxo de atendimento cirúrgico ocorreu nos módulos *Powerchart* e *SurgiNet (*avaliação pré-anestésica e registros cirúrgicos), e no *Saanesthesia* (registro anestésico no intraoperatório).

Por meio da integração entre o RES com o equipamento de anestesia, monitor multiparamétrico e monitor de índice biespectral, via método *Intelligent Input Bus* (IBus), ocorre a migração automática de todos os parâmetros para o RES, a cada 30 segundos. O *Saanesthesia* permitiu a criação de uma interface do estudo, denominada Macro Cirurgia Robótica Projeto AVATaR, por meio da habilitação para o registro de dados pertinentes ao estudo e que não eram habitualmente computados. O anestesista habilitava a ferramenta no início do procedimento (Figura 2).

**Figura 2:** Tela inicial da Macro Cirurgia Robótica Projeto *Assessment of Ventilatory Management During General Anesthesia for Robotic Surgery and Its Effects on Postoperative Pulmonary Complications* (AVATaR)

IOT: intubação orotraqueal.

É importante enfatizar que o RES utilizado para a coleta dos dados é rotineiramente utilizado no HIAE para o cuidado clínico habitual dos pacientes, tendo sido submetido a todos os processos de segurança requeridos pelo hospital. Ainda, ele foi validado previamente ao início do estudo e segue as normas do *ONC Health IT Certification Program*. Ainda, o RES não permite a adulteração de dados; qualquer correção de informação é realizada sobre a forma de adendos, e nenhuma informação é apagada − apenas acrescentada. Por fim, os dados contidos no RES fazem parte do prontuário do paciente e seguem as normas legais vigentes para tal documentação. Todas as informações obtidas por meio do RES foram registradas no CRF em papel que, em conjunto com o RES, é o documento fonte do paciente, conforme autorização da auditoria em pesquisa da instituição. Os documentos em papel estão armazenados com os pesquisadores responsáveis pelo estudo.

### Planejamento do uso secundário do suporte eletrônico na coleta de dados

O CRF final do estudo era composto por três CRF, de acordo com as fases do perioperatórias, tendo sido já descritos acima. A coleta de dados foi delineada em seis etapas (Figura 3):

**Figura 3:** Fluxo de desenvolvimento do sistema para coleta de dados

CRF: ficha de coleta de dados; AVATaR: *Assessment of Ventilatory Management During General Anesthesia for Robotic Surgery and Its Effects on Postoperative Pulmonary Complications.*

1. Cruzamento das informações de registro habitual no RES com o CRF.
2. Desenvolvimento da Macro AVATaR, para coleta automática dos dados no intraoperatório, junto ao setor de Tecnologia da Informação (Figura 4).
  **Figura 4:** Tela de coleta de dados da Macro Cirurgia Robótica Projeto *Assessment of Ventilatory Management During General Anesthesia for Robotic Surgery and Its Effects on Postoperative Pulmonary Complications* (AVATaR)
3. Formulação de um documento para o registro manual das informações do CRF do estudo não recuperáveis pelo RES.
4. Treinamento dos colaboradores para a aplicação do TCLE, preenchimento dos dados de coleta manual e sobre a ferramenta Macro AVATaR.
5. Avaliação diária da qualidade dos dados pelo pesquisador principal, por meio de visita ao paciente, e consulta ampla ao RES.
6. Preenchimento do CRF eletrônico no sistema *Research Electronic Data Capture* (REDCap), pelo pesquisador principal.

### Características da ficha de coleta de dados eletrônico do estudo e do manejo dos dados

Todos os dados coletados são incluídos no CRF eletrônico final do estudo no REDCap^®^ por meio da Internet. O formulário encontra-se hospedado nos servidores do HIAE em um ambiente seguro e controlado para garantir a confidencialidade. O sistema tem as seguintes funções: registro de pacientes, entrada de dados, limpeza de dados, trilhas de auditoria e exportação de dados para análise estatística.

Todos os dados inseridos passam por um gerenciamento periódico. O objetivo do plano de gerenciamento de dados clínicos é fornecer dados de alta qualidade, adotando procedimentos padronizados para minimizar o número de erros e dados perdidos e, consequentemente, gerar um banco de dados preciso para a análise. O monitoramento remoto é realizado para sinalizar padrões aberrantes, problemas com consistência, credibilidade e outras anomalias, de acordo com consultas predefinidas criadas no sistema. Quaisquer valores de dados ausentes e discrepantes são revistos individualmente e completados ou corrigidos, sempre que possível.

## RESULTADOS

### Quantificação dos dados coletados

Foram incluídos 94 pacientes em 30 dias do estudo. No CRF-1, das 34 variáveis, 26 (76,5%) foram obtidas eletronicamente. No CRF-2, 214 de 224 variáveis (95,5%) foram obtidas eletronicamente. No CRF-3, todos os dados (100%), de 12 a 57 variáveis, a depender do tempo de internação hospitalar, foram obtidos eletronicamente.

### Qualidade dos dados

No CRF-1, em 15 casos (15,6%), houve necessidade de completar os dados por meio da visita ao paciente pelo pesquisador principal. Em três pacientes (3,1%), houve troca do diagnóstico de ausência de “tabagismo prévio” para “ex-tabagista”. Na coleta de dados para o CRF-2, ocorreu falha total da migração automática dos dados em três pacientes, e a causa foi a não sincronização entre os dispositivos de monitorização e o *Saanesthesia,* por desconhecimento no manuseio da ferramenta por parte do anestesista. Houve falha parcial na coleta de dados em somente cinco pacientes (5,2%). Não houve necessidade de correção de nenhum dos dados após a obtenção eletrônica deles. Na coleta do CRF-3, não houve necessidade de correção de qualquer dado.

## DISCUSSÃO

No presente estudo, descrevemos uma metodologia de automação de coleta de dados para pesquisa clínica utilizando um RES. Foi encontrada variabilidade no percentual de preenchimento dos CRF com dados obtidos do RES. Entre as fichas coletadas, o CRF-1 foi o que apresentou menor quantidade de dados capazes de serem obtidos eletronicamente. O grau de intersecção entre as informações necessárias para o preenchimento do CRF do estudo com aquelas contidas obrigatoriamente no RES motivou essas variações de desempenho. Por exemplo, no CRF-3, todas informações pertinentes ao estudo já estavam disponíveis naturalmente no RES, ao passo que, no CRF-1, existia quantidade maior de dados que não faziam parte do registro habitual do anestesista e, por isso, tiveram que ser obtidos manualmente.

A participação do RES na pesquisa clínica tem diversas aplicações, indo desde o uso secundário dos RES no suporte a pesquisa clínica,^6^ até o *Registry-based randomized controlled trials*, que são estudos inteiramente suportados pelos RES.^7^ Entre os potenciais benefícios, podemos destacar a redução de custos, o rápido recrutamento de pacientes, os dados gerados em situação de “vida real” e o potencial seguimento completo dos pacientes.^7^ As considerações éticas são desafios importantes.^8^

A avaliação da qualidade dos dados obtidos por meio do RES é um desafio e a ausência de dispositivos que possam assegurar a validade do uso desta ferramenta no auxílio à pesquisa pode ser uma fonte de viés.^9,10^ Estudo prospectivo prévio avaliou o potencial do uso do RES como método substitutivo para a coleta de dados de 358 pacientes no período pós-operatório e evidenciou que mais de 96% dos dados necessários foram completamente preenchidos pelo RES, mas a taxa de concordância entre as informações avaliadas apresentou variações de 19 a 73%. Desta forma, o autor ressalta a necessidade da avaliação variável a variável para o planejamento desta metodologia de obtenção de dados para o estudo clínico.^11^

Sob diversos aspectos, esta foi uma solução inovadora, pois apropriou-se das potencialidades de um RES que não foi primariamente desenvolvido para a pesquisa clínica e possibilitou a realização da coleta de dados e da anestesia pelo mesmo profissional, sem prejuízo para qualquer dos processos. A ampla adesão dos colaboradores foi resultado da mínima interferência da coleta de dados sobre o manejo anestésico. Além disso, a ferramenta auxiliou na produção de dados fidedignos e passíveis de conferência, o que pode evitar manipulações, fraudes e ocorrência de publicações de retratação científica.^12^ Em um cenário em que o descrédito acerca da veracidade dos trabalhos científicos é objeto de publicações,^13^ a tecnologia pode ser um importante aliado, mas um pesquisador com função ativa ainda não pode ser dispensado, pois permite identificar e corrigir falhas no processo.

## CONCLUSÃO

Este estudo descreve metodologia eficaz para o uso secundário do Registros Eletrônicos em Saúde na coleta de dados para pesquisa e demonstra a existência de sobreposição entre os dados coletados na prática clínica e aqueles coletados para um grande estudo clínico. O auxílio da coleta de dados com esta ferramenta tem potencial de minimizar o trabalho manual e, consequentemente, aumentar a adesão dos colaboradores e a qualidade dos dados obtidos. Ainda, por meio da dupla checagem de todas as variáveis, verificamos que este método foi capaz de produzir dados confiáveis e de alta qualidade. Desta forma, entendemos que, por meio de um planejamento cuidadoso, a utilização do Registros Eletrônicos em Saúde como auxílio para coleta de dados em estudos clínicos tem grande potencial.
